# Reduction of lymphotoxin beta receptor induces cellular senescence via the MDMX-p53 pathway

**DOI:** 10.1038/s41420-025-02708-1

**Published:** 2025-08-29

**Authors:** So Young Kim, Bin Lee, Je-Jung Lee, Man Sup Kwak, Woo Joong Rhee, In Ho Park, Jeon-Soo Shin

**Affiliations:** 1 Department of Microbiology, Yonsei University College of Medicine, Seoul, South Korea; 2 Brain Korea 21 FOUR Project for Medical Science, Yonsei University College of Medicine, Seoul, South Korea; 3 Institute for Immunology and Immunological Diseases, Yonsei University College of Medicine, Seoul, South Korea; 4 Department of Biomedical Sciences, Yonsei University College of Medicine, Seoul, South Korea

**Keywords:** Ubiquitylation, Senescence

## Abstract

The lymphotoxin β receptor (LTβR), a key activator of non-canonical NF-κB signaling, is expressed in various cells, including cancer cells. Although high expression of LTβR has been associated with poor patient prognosis and drug resistance, conflicting evidence suggested that LTβR induces apoptosis. To investigate the functional role of LTβR in tumors, we performed LTβR knockdown in cancer cells. We found that LTβR knockdown induced senescence phenomena such as reduced cell number; increased cell size; increased SA-β-Gal activity; and upregulated p53, MDM2 and p21 expression. Moreover, LTβR knockdown induced p21-mediated senescence in p53 WT cancer cells, but not in p53 mutant cancer cells. The level of p53 is regulated by MDM2 and MDMX; MDMX enhances MDM2 activity but is also subject to MDM2-mediated degradation in the nucleus. We found that the intracellular domain of LTβR bound to MDMX thereby inhibited its nuclear translocation, which in turn reduced MDMX ubiquitination and consequently promoted p53 ubiquitination. Additionally, tumors derived from B16F10^LTβR-KO^ cells in WT mice exhibited significantly reduced growth compared to those derived from B16F10^WT^ cells. These results demonstrate that LTβR regulates p53 protein levels by modulating MDMX stability and localization, resulting in p53-mediated cellular senescence.

**Figure:**
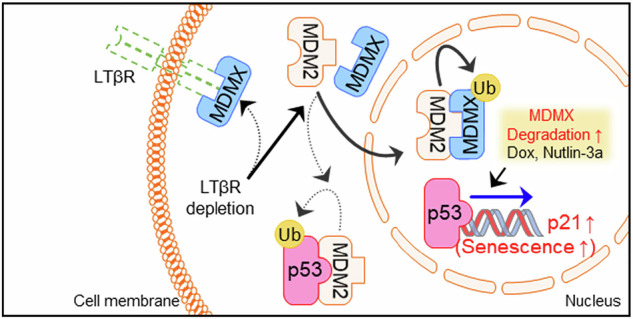
**LTβR regulates p53-mediated senescence by inhibiting MDMX nuclear translocation and degradation**. LTβR interacts with MDMX in the cytoplasm, preventing its nuclear translocation and degradation under normal conditions (dotted arrows). When LTβR is depleted, MDMX is translocated into the nucleus by MDM2, and undergoes degradation (solid arrows). This reduces p53 degradation and consequently activates p53, leading to p21 transcription and the induction of cellular senescence. Treatment with doxorubicin (Dox) or nutlin-3a further enhances p53-mediated transcriptional activation of p21, and their combination with LTβR depletion exerts an additive effect in promoting cellular senescence.

**LTβR regulates p53-mediated senescence by inhibiting MDMX nuclear translocation and degradation**. LTβR interacts with MDMX in the cytoplasm, preventing its nuclear translocation and degradation under normal conditions (dotted arrows). When LTβR is depleted, MDMX is translocated into the nucleus by MDM2, and undergoes degradation (solid arrows). This reduces p53 degradation and consequently activates p53, leading to p21 transcription and the induction of cellular senescence. Treatment with doxorubicin (Dox) or nutlin-3a further enhances p53-mediated transcriptional activation of p21, and their combination with LTβR depletion exerts an additive effect in promoting cellular senescence.

## Introduction

The lymphotoxin beta receptor (LTβR), also known as tumor necrosis factor receptor superfamily member 3 (TNFRSF3), is a well-studied molecule in immunology [1–6]. Studies on mice deficient in LTβR have exhibited significant defects in the development and formation of secondary lymphoid organs, including lymph nodes [7]. As a receptor protein, LTβR induces non-canonical NF-κB signaling [8–10]. Upon stimulation and internalization, LTβR recruits TRAF2 and TRAF3, which are subsequently degraded by cIAP1/2. This leads to the cleavage of the p100 precursor bound to RelB, forming the RelB–p52 heterodimer. Furthermore, this signaling is implicated in apoptosis through the LTβR–TRAF2–cIAP1-Smac signaling pathway [11] and cIAP1/2–IKKα/β-mediated canonical NF-κB signaling [12].

Although LTβR signaling activation is known to induce apoptosis [10, 11, 13, 14], some studies have shown a reduced apoptosis rate [15, 16]. Moreover, elevated LTβR expression in several cancer types has been associated with poor patient prognosis [17–20]. These findings suggest LTβR contributes to cancer development and progression, as transfection with truncated or full-length LTβR can result in carcinogenesis [21]. In addition, data from the Cancer Therapeutics Response Portal (CTRP) database of the Broad Institute indicate that LTβR expression is negatively correlated with the efficacy of certain anti-cancer drugs, including doxorubicin (topoisomerase II inhibitor) and nutlin-3a (MDM2 inhibitor). Given the lack of clear mechanism explaining how LTβR affects drug resistance and apoptosis, we aimed to elucidate this gap using an LTβR knockdown system in melanoma cells, where high LTβR expression is correlated with poor survival rate.

The p53 protein is a prominent tumor suppressor and transcription factor [22]. Its expression is induced in response to DNA damage, triggering the expression of various downstream proteins, including MDM2. In turn, MDM2 is a ubiquitin E3 ligase that regulates p53 turnover [23]. Under normal conditions, p53 is maintained at low levels by MDM2. Additionally, MDM2 collaborates with MDMX, a structurally similar protein that lacks E3 ligase activity, to suppress p53. MDMX relies on MDM2 for nuclear localization owing to the lack of nuclear localization signal. MDM2 binds to the MDMX RING domain to facilitate its nuclear import [24]. Following DNA damage, MDMX is translocated to the nucleus by MDM2, where it undergoes degradation, leading to an upregulation of p53. Inhibiting MDMX nuclear translocation results in MDMX accumulation and subsequently leads to the downregulation of p53, MDM2, and p21 [25]. These findings highlight the importance of MDMX localization in regulating p53 degradation. However, how MDMX is stabilized in the cytoplasm remains unclear.

The p53 protein is a key regulator of p21 expression, a cyclin-dependent kinase (cdk) inhibitor that induces G1/S cell cycle arrest and contributes to cellular senescence [26, 27]. Cellular senescence can be induced by various cellular stress stimuli, such as DNA damage, reactive oxygen species (ROS), and oncogene activation [28]. Senescent cells exhibit distinct morphological changes, including increased cell size, a flattened shape, elevated senescence-associated β-Galactosidase (SA-β-Gal) activity, and higher levels of proteins such as p21 [29–31]. Induction of cellular senescence can be used as a promising strategy in cancer therapy, particularly for halting the growth of cancer cells resistant to apoptosis [32, 33].

In this study, we found that LTβR knockdown induces senescence in cancer cells, which is mediated by the upregulation of p53 and p21 expression. We propose that LTβR regulates p53 activity by preventing MDMX nuclear translocation and degradation. These findings provide new insights into the potential of LTβR as a therapeutic target in cancer.

## Results

### Knockdown of LTβR induces cellular senescence

While the role of LTβR as a receptor is well-documented, recent studies have linked high LTβR expression with poor prognosis in various cancers [20], suggesting its critical role in key cellular processes. To investigate the impact of LTβR knockdown on cellular phenotype, we used specific siRNAs to knock down LTβR expression for 48 h in A375, A549, B16F10, and J774 cell lines (Fig. S1A). LTβR knockdown led to increased cell size and a reduced cell number (Fig. S1B, C). Immunofluorescence staining revealed decreased expression of Ki67, a proliferation marker, in LTβR knockdown cells (Fig. S1D). Further analysis showed an increased proportion of cells in the G1 phase and elevated SA-β-Gal activity, which was detected using the senescence green probe (Fig. S1E, F). Together, these results suggest that LTβR knockdown may induce cellular senescence, which is characterized by growth arrest and apparent morphological changes.

### Depletion of LTβR induces p53-mediated senescence

**Fig. 1:**
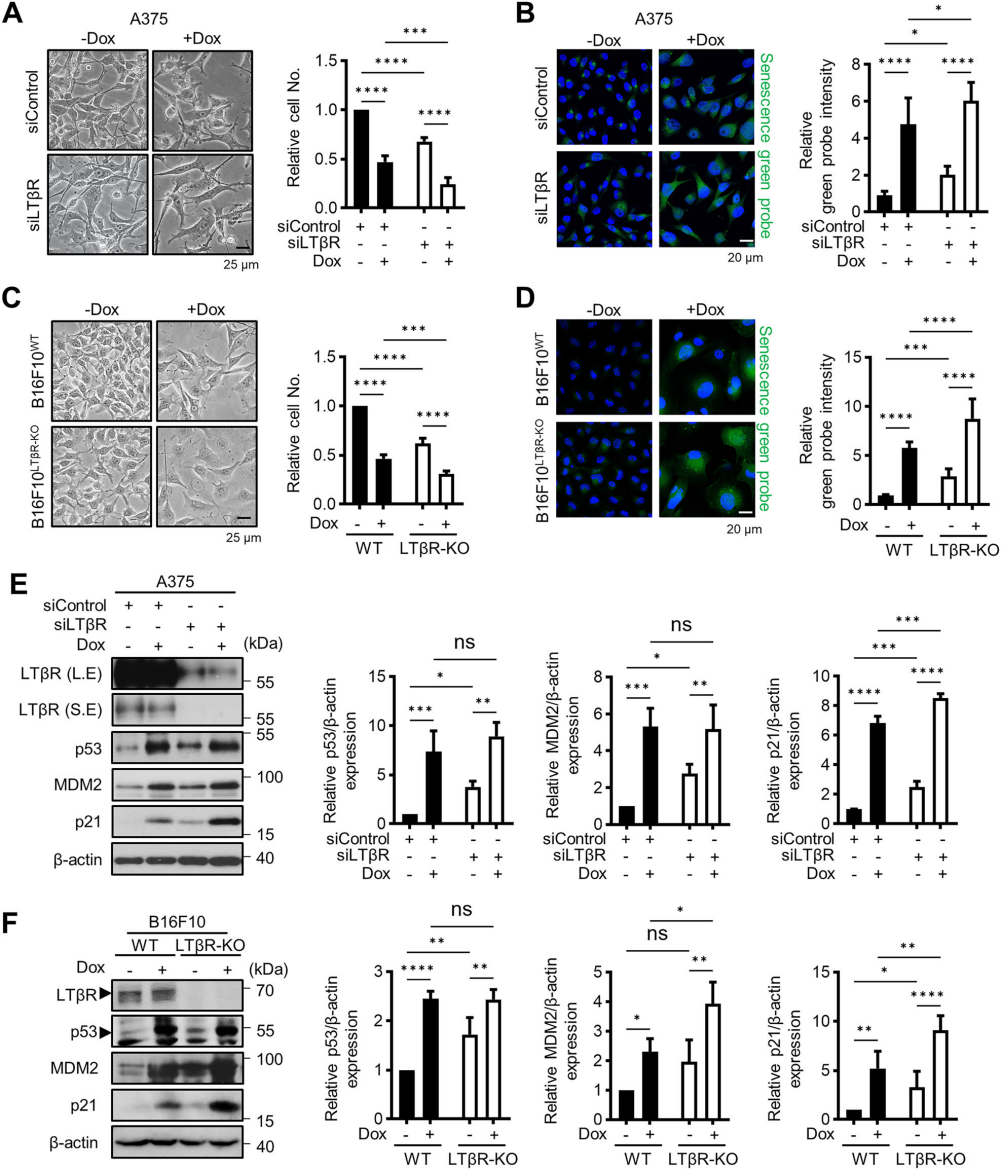
Depletion of LTβR induces p53-mediated senescence. **A**, **B** A375 cells were transfected with 100 nM of siControl (control siRNA) or siLTβR (LTβR siRNA), followed by 100 ng/ml Dox treatment for 48 h. Morphological changes, relative cell number (**A**), and confocal images of a senescence green probe (**B)** were analyzed. **C**, **D** B16F10^WT^ (LTβR WT), and B16F10^LTβR-KO^ (LTβR knockout) cells were treated with 100 ng/ml Dox for 48 h. Morphological changes and relative cell number (**C**), and confocal images of senescence green probe (**D)** were examined. **E**, **F** Western blot images of A375 and B16F10 cells showing the indicated proteins in LTβR-depleted cells. S.E. short exposure, L.E. long exposure. Band intensities of p53, p21 and MDM2 were measured using ImageJ and normalized to β-actin. Results are presented as the mean ± SD from three separate experiments. **B**, **D** Fluorescence intensities for relative senescence green probe were quantified by ImageJ, and data are shown as mean ± SD from three independent experiments (*n* = 3). *p < 0.05, **p < 0.01, ***p < 0.001, ****p < 0.0001, using Fisher’s LSD post hoc test. n.s not significant.

Among the many pathways that drive cellular senescence, we focused on exploring the well-established p53-mediated senescence pathway. To confirm whether the senescent phenotype observed in LTβR knockdown cells is related to p53-mediated pathways, we used doxorubicin (Dox), a well-known inducer of senescence, as a positive control. While Dox-treated cells exhibited reduced cell numbers and increased SA-β-Gal activity, LTβR knockdown in A375 cells similarly led to decreased cell numbers and elevated SA-β-Gal activity, which showed an additive effect when combined with Dox treatment (Fig. 1A, B). To rule out transient effects of siRNA, we prepared LTβR knockout (KO) B16F10 (B16F10^LTβR-KO^) cells using the CRISPR/Cas9 system. Comparable results were also observed in B16F10^LTβR-KO^ cells (Fig. 1C, D), suggesting that the LTβR depletion induces senescence. Given that p53 is a pivotal regulator of cellular senescence, p53 protein levels were examined (Fig. 1E, F). We observed increased levels of p53, along with elevated levels of MDM2, a key regulator of p53 that is also known to be upregulated during p53-mediated senescence rather than apoptosis [34, 35]. Moreover, we observed an upregulation of p21, a key mediator of p53-mediated cell cycle arrest, in LTβR-depleted cells.

These phenomena were consistent in normal human lung fibroblast IMR90 cells (Fig. S2A–C). However, in the p53 mutant human colorectal cancer cell line HT-29, which lacks p53 transcription activity, senescence was induced by Dox but not by LTβR knockdown, underscoring the role of p53 in LTβR depletion-induced senescence. (Fig. S2D–F). These findings suggest that LTβR regulates cellular senescence through a p53-dependent pathway.

### LTβR overexpression attenuates senescence

**Fig. 2:**
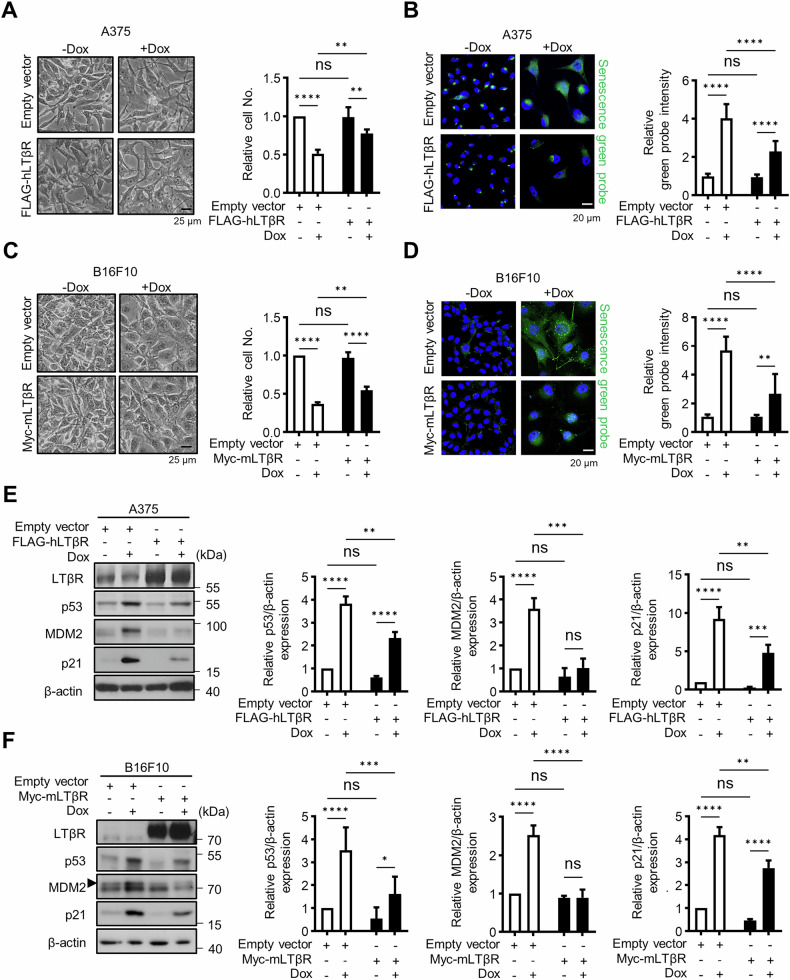
LTβR inhibits senescence. **A**–**D** A375 and B16F10 cells were transfected with LTβR plasmid followed by 100 ng/ml Dox treatment for 48 h. Cells were photographed for analyzing morphological change and relative cell number (**A**, **C**), and stained using a senescence green probe (**B**, **D**). Relative fluorescence intensity for the senescence green probe was quantified using ImageJ. **E**, **F** Western blot images of A375 and B16F10 cells for the indicated proteins are representative of three experiments, and the relative p53, p21, and MDM2 protein levels were measured. Bands were quantified using ImageJ software and normalized to β-actin. Graphical data are represented as mean ± SD from three independent experiments (*n* = 3). *p < 0.05, **p < 0.01, ***p < 0.001, ****p < 0.0001, using Fisher’s LSD post hoc test. n.s, not significant.

Next, to investigate the effects of LTβR overexpression on cellular senescence and p53 activity, A375 and B16F10 cells were transfected with either an empty vector plasmid or an LTβR-expressing plasmid, followed by treatment with Dox. LTβR-overexpressing cells showed a less pronounced reduction in cell number and increase in SA-β-Gal activity compared to control cells when treated with Dox (Fig. 2A–D). Furthermore, western blot analysis revealed lower levels of p53, MDM2, and p21 in LTβR-overexpressing cells compared to controls (Fig. S3A), suggesting that LTβR overexpression reduces Dox-induced senescence in a p53-dependent manner (Fig. 2E, F). To further validate the effect of LTβR overexpression in cells, we restored LTβR expression in LTβR KO cells by transfecting them with an LTβR-expressing vector (Fig. S3B–D). Restored LTβR expression attenuated the senescent phenotype, as shown by increased cell numbers, decreased SA-β-Gal activity, and reduced levels of p53, p21, and MDM2 under Dox treatment. These results indicate that LTβR overexpression can suppress Dox-induced senescence.

### LTβR modulates p53 by regulating MDMX protein degradation

**Fig. 3:**
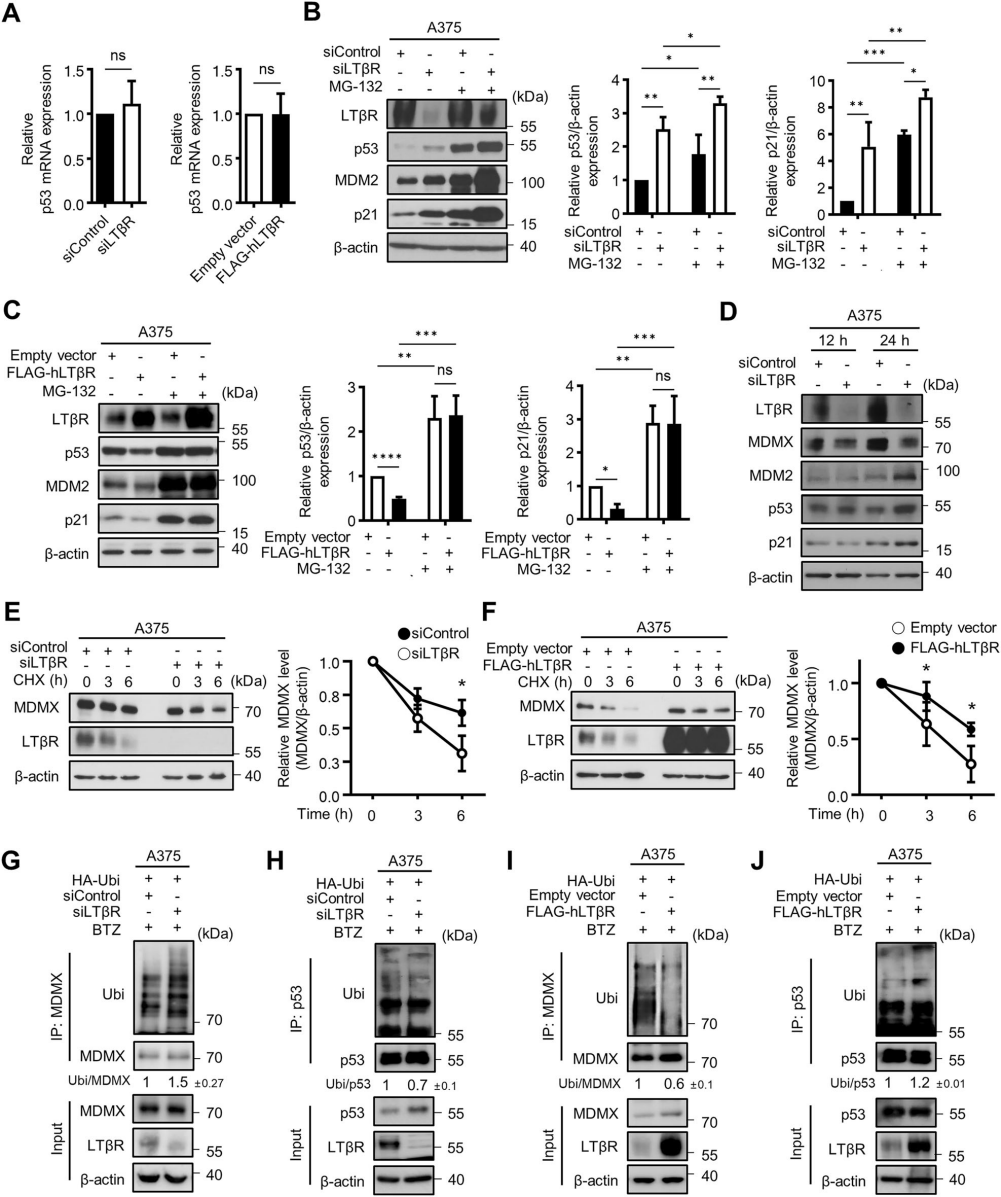
LTβR modulates p53 and MDMX protein expression. **A** Relative p53 mRNA levels in A375 cells with LTβR knockdown or LTβR-overexpressing cells determined by real-time PCR. **B**, **C** Western blot analysis of p53 and p21 protein levels in A375 cells after 20 μM of MG-132 treatment for 4 h, following siRNA (**B)** or LTβR plasmid (**C)** transfection. p53 and p21 relative band intensities were quantified using ImageJ software, and normalized to β-actin. **D** Western blot analysis of MDMX, p53, MDM2, and p21 protein expression in A375 cells after 12 h or 24 h of siControl or siLTβR transfection. **E**, **F** Cycloheximide (CHX, 100 μg/ml) chase assays were conducted to determine MDMX protein stability in LTβR-overexpressing and LTβR knockdown cells for the indicated time and quantified using ImageJ. **G**–**J** A375 cells were treated with 80 nM of BTZ for 4 h, and whole cell lysate was subjected to immunoprecipitation to confirm MDMX ubiquitination and p53 ubiquitination. Relative expression levels of ubiquitinated MDMX and p53 were measured using ImageJ software, normalized, and presented as mean ± SD from three independent experiments. Graphical data are presented as means ± SD from three independent experiments (*n* = 3). n.s not significant, using an unpaired Student’s *t*-test (**A**). *p < 0.05, **p < 0.01, ***p < 0.001, ****p < 0.0001, using Fisher’s LSD post hoc test (**B**, **C)** or Šidák’s multiple comparison test (**E**, **F**). n.s not significant.

To determine whether LTβR influences p53 transcription, p53 transcription levels in LTβR knockdown and overexpressing cells were analyzed using real-time PCR. No significant changes were observed in p53 mRNA levels (Fig. 3A). However, p21 mRNA, a downstream target of p53, was significantly upregulated, suggesting post-transcriptional regulation of p53 by LTβR (Fig. S4A). These results align with previous RNA-seq data showing increased cdkn1a (p21) levels in hematopoietic stem cells of LTβR KO mice [36] (Fig. S4B). Treatment with the proteasome inhibitor MG-132 further elevated p53 levels in LTβR knockdown cells and restored p53 and p21 levels in LTβR-overexpressing cells (Fig. 3B, C). This indicates that knockdown of LTβR prevents degradation of the p53 protein. As p53 protein degradation is regulated by MDM2 and MDMX, we assessed their expression in LTβR knockdown cells. MDMX protein level declined at 12 h after LTβR siRNA transfection, while no significant changes were observed in the level of p53, MDM2, and p21 compared to control cells before 24 h (Fig. 3D and S4C). These findings indicate that the expression of p53, MDM2, and p21 may be influenced as a consequence of changes in MDMX. Knockdown of MDMX increased p53, MDM2, and p21 levels without affecting LTβR expression (Fig. S4D), suggesting that LTβR regulates MDMX, which in turn inhibits p53 degradation. To further support the role of LTβR in p53 protein degradation, A375 cells were treated with MDM2 inhibitor nutlin-3a, which disrupts the MDM2–p53 binding and induces p53-mediated cellular senescence. MDMX overexpression has been reported to counteract the effect of nutlin-3a by preventing p53 activation [37, 38]. In LTβR knockdown cells, p53 levels increased rapidly upon nutlin-3a treatment (Fig. S4E), whereas LTβR-overexpressing cells exhibited a delayed increase (Fig. S4F).

To examine whether LTβR regulates MDMX protein degradation, A375 cells were treated with the protein synthesis inhibitor cycloheximide (CHX). MDMX protein levels decreased more rapidly in LTβR knockdown cells and more slowly in LTβR-overexpressing cells (Fig. 3E, F), indicating that LTβR stabilizes MDMX protein. Finally, changes in MDMX and p53 ubiquitination patterns were observed in both LTβR knockdown and LTβR-overexpressing cells (Fig. 3G–J). Ubiquitination of MDMX was increased in LTβR knockdown cells and decreased in LTβR-overexpressing cells, whereas p53 ubiquitination showed the opposite pattern—decreased in LTβR knockdown cells and increased in LTβR-overexpressing cells. These results suggest that LTβR negatively regulates MDMX ubiquitination, thereby promoting p53 degradation.

### LTβR interacts with MDMX in cytosol

**Fig. 4:**
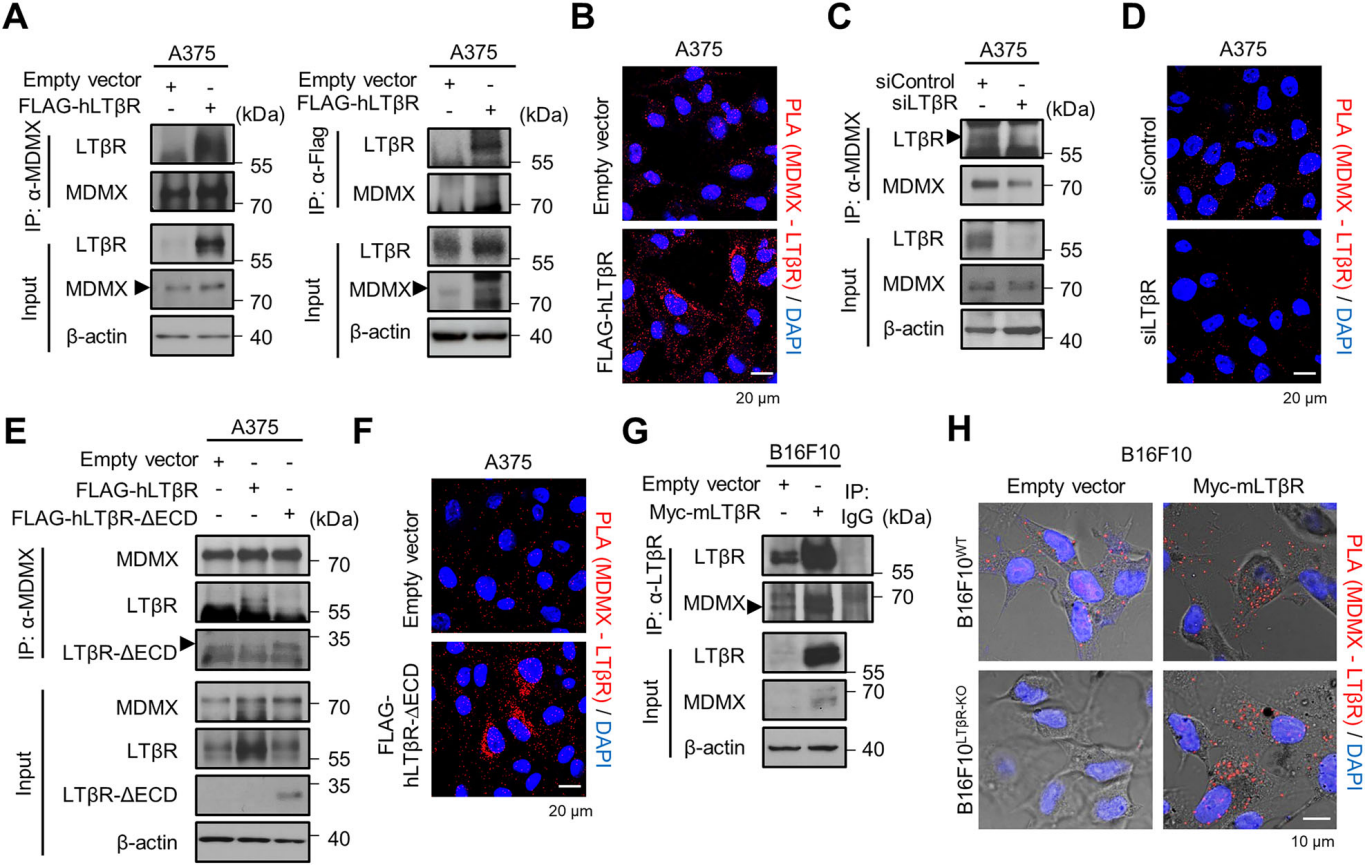
LTβR interacts with MDMX in the cytosol. **A**, **B** A375 cells were transfected with LTβR plasmid for 48 h. LTβR-overexpressing cells were subjected to immunoprecipitation with LTβR and MDMX, and PLA was performed using MDMX, Flag, and LTβR antibodies. **C**, **D** A375 cells were transfected with 100 nM of siControl or siLTβR for 48 h, and subjected to immunoprecipitation and PLA using LTβR and MDMX antibodies. **E**, **F** Immunoprecipitation and PLA of extracellular domain (ECD)-deleted LTβR-transfected cells were performed using MDMX and LTβR antibodies. **G** Immunoprecipitation of LTβR and immunoblotting using MDMX antibody validated the interaction in LTβR-overexpressing B16F10 cells. **H** B16F10^WT^ and B16F10^LTβR-KO^ cells were transfected with LTβR plasmid, and PLA was performed to determine the location of interaction between LTβR and MDMX.

We hypothesized that LTβR stabilizes the MDMX protein by binding to it, as LTβR contains an α-helix near its intracellular TRAF-binding domain and MDMX has a Zn²⁺ finger-like domain in its MDM2-binding region. To assess the potential interaction, we used the HADDOCK 2.4 web server to predict the protein–protein docking score. The computational analysis of HADDOCK score for LTβR α-helix–MDMX–MDM2 binding site was −66.1 ± 2.2 (cluster size 21, Z score −1.3), suggesting high probability of binding [39, 40]. To confirm the interaction between LTβR and MDMX, LTβR knockdown and overexpressing cells were subjected to immunoprecipitation and proximity ligation assays (PLA). LTβR-overexpressing cells showed increased LTβR–MDMX binding, whereas LTβR knockdown cells exhibited reduced interaction (Fig. 4A–D), supporting an interaction between LTβR and MDMX. To test whether this binding occurs through the intracellular domain, we generated a truncated form of LTβR (ΔECD; Δ1-227 aa), which lacks the extracellular domain but retains the transmembrane and intracellular regions. Both immunoprecipitation and PLA demonstrated that LTβR-∆ECD still interacts with MDMX, suggesting that the interaction occurs in the cytoplasm (Fig. 4E, F). We also observed increased interaction in LTβR-overexpressing B16F10 cells, as well as restored PLA signal in LTβR-overexpressing B16F10^LTβR-KO^ cells (Fig. 4G, H), confirming that this interaction also occurs in mouse cells.

It has been shown that overexpression of LTβR activates non-canonical NF-κB signaling through self-oligomerization, independent of its extracellular domain [41–44]. To investigate whether the extracellular domain is required to attenuate cellular senescence, we transfected A375 cells with LTβR-ΔECD, a truncated form lacking the extracellular domain. The results were consistent with those of full-form LTβR overexpression, indicating that the effects are independent of extracellular domain (Fig. S5A–C).

Next, A375 cells were treated with LIGHT protein (a LTβR ligand) (Fig. S6A–D) to examine whether extracellular signaling contributes to p53-mediated cellular senescence. LIGHT treatment resulted in increased levels of IκBα and decreased levels of LTβR, consistent with previous research showing that ligand-induced endocytosis of LTβR limits canonical NF-κB signaling and promotes its degradation [44]. Although LIGHT treatment reduced LTβR expression, MDMX protein levels remained unaffected, which is likely due to decreased levels of MDM2, a MDMX-degrading enzyme. We observed decreased levels of p53 and MDM2 in LIGHT-treated cells, suggesting that p53 and MDM2 levels might be regulated through LTβR-dependent NF-κB signaling. LIGHT treatment led to a comparable increase in p21 levels in both control and LTβR-overexpressing cells, in contrast to the results observed in our overexpression model. The SA-β-Gal activity assay further suggests that LIGHT does not significantly affect the senescence state in LTβR knockdown cells, but induces minor changes in LTβR-overexpressing cells (Fig. S6B, D). To examine whether LIGHT treatment affects the interaction between LTβR and MDMX, we treated cells with LIGHT for 4 h (Fig. S6E). However, the interaction appeared to be primarily regulated by the expression levels of LTβR following LIGHT treatment. Taken together, p53, MDM2, and p21 may be influenced by NF-κB signaling, while MDMX–p53-mediated cellular senescence is likely associated with the expression of LTβR.

### LTβR inhibits MDMX nuclear translocation

**Fig. 5:**
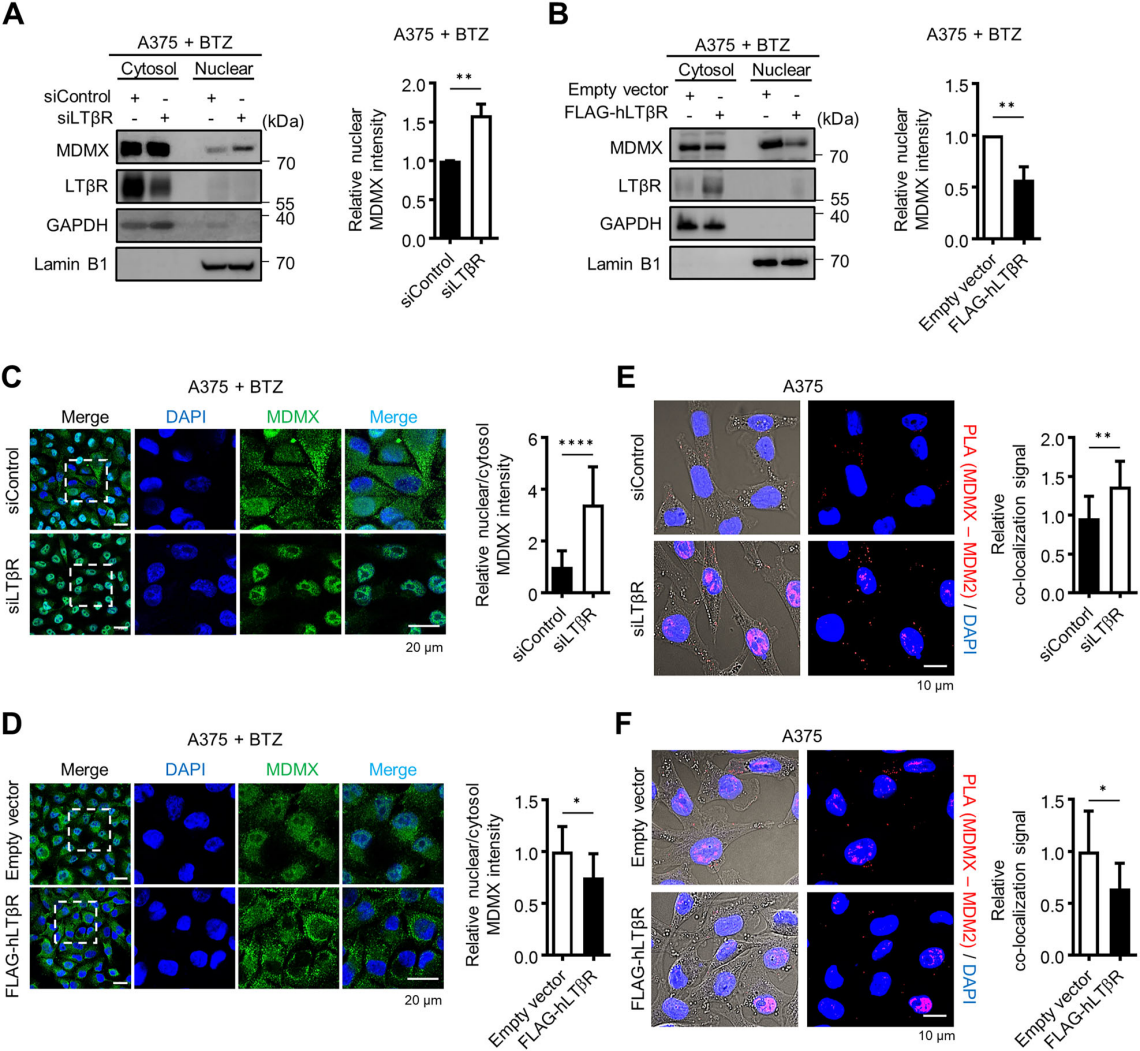
LTβR inhibits MDMX nuclear translocation. **A**, **B** A375 cells were treated with 80 nM BTZ (bortezomib) for 4 h after siRNA or plasmid transfection. Nuclear and cytosol fractions were isolated, followed by western blotting. GAPDH (cytosol) and Lamin B1 (nuclear) were used as loading controls. **C**, **D** Confocal microscopy was used to assess MDMX localization. Nuclear MDMX band and fluorescence intensities were measured relative to cytosolic MDMX using ImageJ. **E**, **F** PLA was performed to confirm the relative interaction between MDM2 and MDMX in LTβR knockdown cells or LTβR-overexpressing cells. Relative co-localization signals of MDM2 and MDMX (shown in graph) were quantified using ImageJ. Graphical data are presented as means ± SD (*n* = 3) from three independent experiments. *p < 0.05, **p < 0.01, ****p < 0.0001, using an un*p*aired Student’s *t*-test.

MDMX lacks a nuclear localization signal and is known to be ubiquitinated in the nucleus by MDM2 [25, 45]. To investigate whether LTβR affects nuclear localization of MDMX, we performed cytosolic and nuclear fractionation following proteasome inhibition using bortezomib (BTZ) to prevent proteasome-mediated degradation of MDMX. Western blot analysis showed that nuclear MDMX levels increased in LTβR knockdown cells but decreased in LTβR-overexpressing cells (Fig. 5A, B). Confocal microscopy results were consistent with the western blot data, showing similar patterns of nuclear MDMX localization (Fig. 5C, D). Comparable results were observed in LTβR KO cells that reconstituted with LTβR (Fig. S7A, B). PLA further confirmed that MDM2–MDMX interaction was enhanced in LTβR knockdown cells but reduced in LTβR-overexpressing cells (Fig. 5E, F). To exclude the potential involvement of the MDMX deubiquitinating enzyme USP7 [46], we performed immunoprecipitation using a USP7 antibody. USP7 has been reported to interact with TRAF6, which in turn modulates NF-κB signaling [47, 48]. Interestingly, the interaction between USP7 and TRAF6 was enhanced following LTβR overexpression, whereas the interaction between USP7 and MDMX showed no significant change upon either LTβR knockdown or overexpression (Fig. S7C, D). These findings suggest that LTβR knockdown induces the nuclear localization of MDMX, by upregulating its interaction with MDM2, which in turn facilitates MDMX degradation in the nucleus and subsequently suppresses the degradation of p53.

### LTβR KO cells delay tumor growth in vivo

**Fig. 6:**
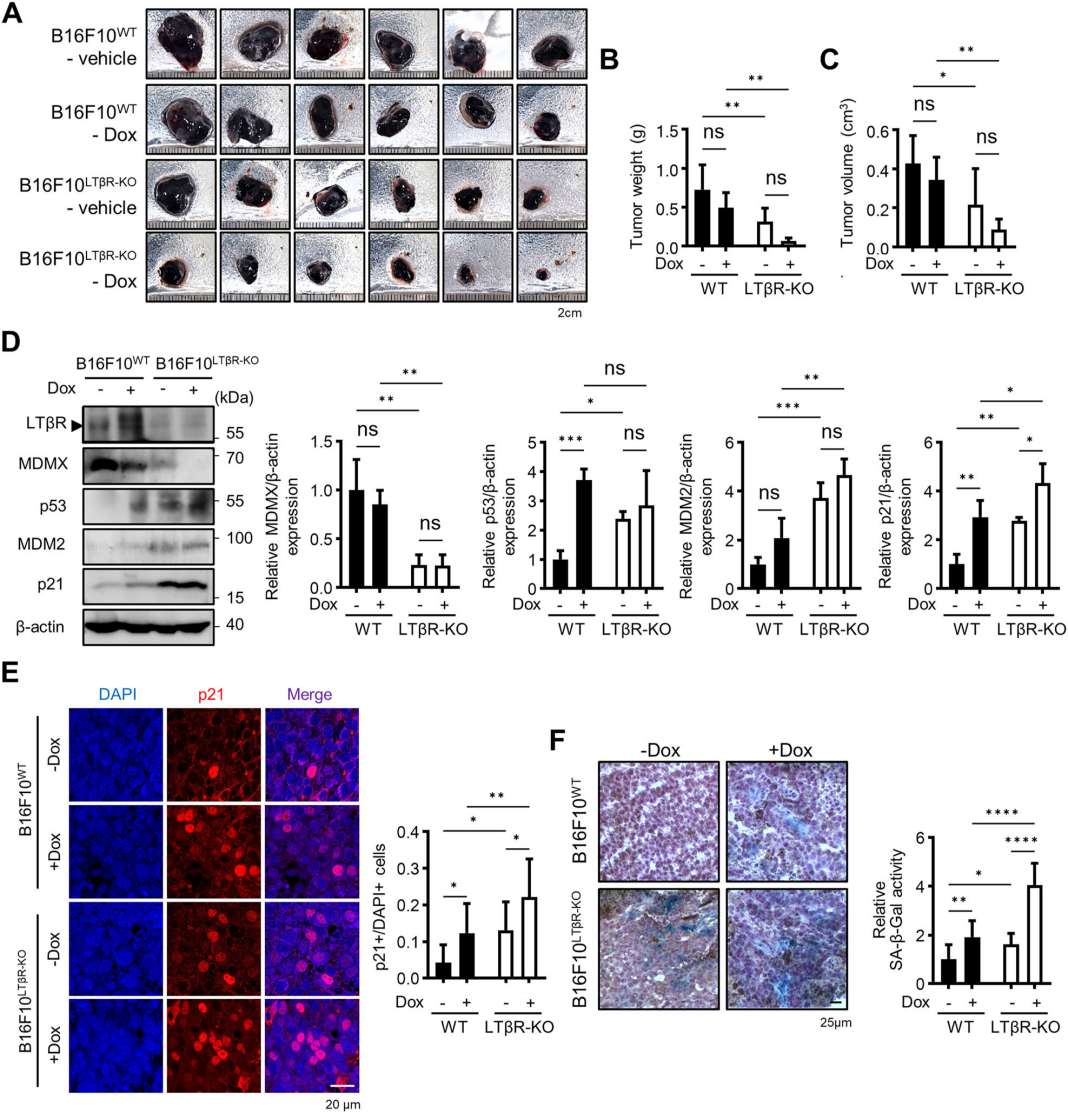
Depletion of LTβR inhibits tumor growth. Tumors were established by the subcutaneously injecting of B16F10^WT^ and B16F10^LTβR-KO^ cells into mice. On day 9 after implantation, mice were administered with 4 mg Dox per kg of mouse body weight. **A**–**C** Tumors were harvested on day 16, photographed, and their weight and volume were measured (*n* = 6). **D** Western blotting of tumor cell lysates for indicated proteins. **E** Paraffin-embedded tissue sections were stained for p21, and relative expression levels were measured using ImageJ. **F** Cryosections of tumor tissue were subjected to SA-β-Gal staining, and relative SA-β-Gal activity was measured using ImageJ. Brown pigments in the histological sections represent melanin deposits. Hematoxylin was used for counterstaining. Graphical data are presented as means ± SD (*n* = 3). *p < 0.05, **p < 0.01, ***p < 0.001, ****p < 0.0001, using Fisher’s LSD post hoc test. n.s not significant.

To investigate the senescence phenotype of LTβR KO cells in WT mice, which express potential ligands such as LIGHT and LTα1β_2_, B16F10^WT^ cells were implanted on the right dorsal side, while B16F10^LTβR-KO^ cells were implanted on the left dorsal side of 8-week-old WT C57/BL6 mice. After 9 days, the mice were treated via intraperitoneal injection with either vehicle (PBS) or Dox (4 mg/kg) to induce a robust synergistic effect on tumor senescence, and were sacrificed 7 days later (Fig. 6A). Tumor measurements indicated that B16F10^LTβR-KO^ tumors were significantly smaller in weight and volume compared with B16F10^WT^ tumors (Fig. 6B, C). Western blot analysis of tumor tissues revealed increased levels of p53, MDM2, and p21 in B16F10^LTβR-KO^ tumors (Fig. 6D). Moreover, fluorescent immunohistochemistry confirmed elevated levels of p21, and cryosection analysis showed higher SA-β-Gal activity in B16F10^LTβR-KO^ tumors, highlighting a pronounced senescence phenotype (Fig. 6E, F).

**Fig. 7:**
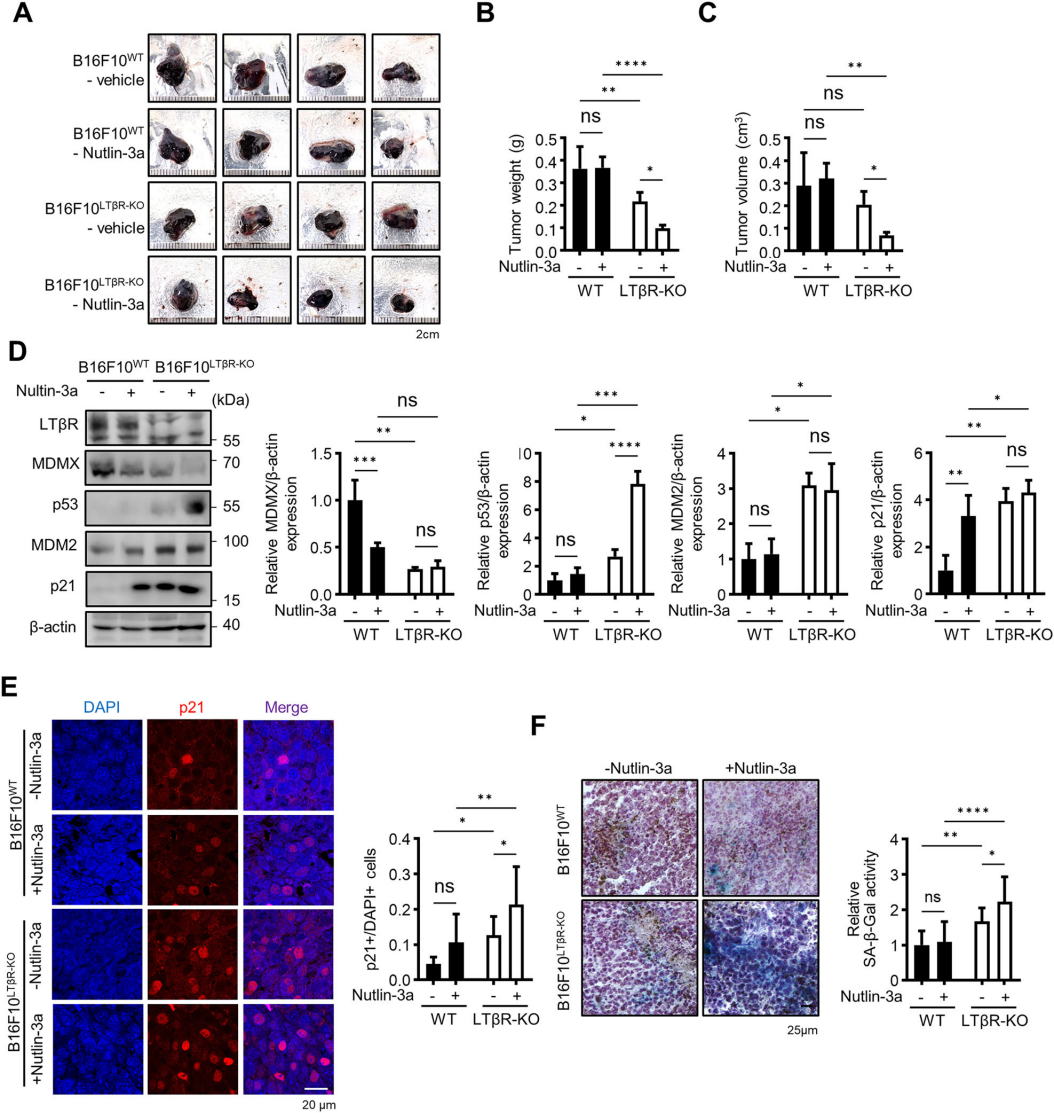
LTβR depletion synergistically enhances inhibition of tumor growth with nutlin-3a. **A**–**C** Tumors were generated by implantating B16F10^WT^ and B16F10^LTβR-KO^ cells into mice. On day 9 after implantation, mice were injected with 20 mg nutlin-3a per kg of mouse body weight. After 7 days, tumors were collected, photographed, and their weight and volume were measured (*n* = 4). **D** Western blot of tumor cell lysates for indicated proteins. **E** Paraffin-embedded tissue sections were stained for p21, and relative expression was measured. **F** Cryosections of tumor tissue were stained with SA-β-Gal to assess senescence activity. Brown pigments in the histological section indicate melanin deposits. Hematoxylin was used for counterstaining. Graphical data are presented as means ± SD (*n* = 3). *p < 0.05, **p < 0.01, ***p < 0.001, ****p < 0.0001, using Fisher’s LSD post hoc test. n.s not significant.

Next, to test the additive effect of MDM2 inhibitor on enhancing p53 activation, mice implanted with B16F10^WT^ and B16F10^LTβR-KO^ cells were treated with nutlin-3a. As shown in Fig.7A–D, nutlin-3a further supported the role of LTβR in regulating p53-mediated senescence through a decrease in tumor growth. Tumor tissue analysis revealed elevated p21 levels and SA-β-Gal staining in nutlin-3a-treated LTβR KO tumors (Fig. 7E, F). These findings indicate that depletion of LTβR delays tumor progression in vivo, suggesting that the combination of LTβR gene targeting and p53-activating drugs may serve as a potential therapeutic strategy for cancer treatment.

## Discussion

LTβR is expressed in various cancer cells, particularly in lung and skin cancers [20]. LTβR stimulation with its ligand LIGHT, or agonist antibodies, as well as overexpression induces apoptosis in cells [17–19, 49]. Despite its known role in inducing apoptosis, the reason for LTβR overexpression in certain cancers remains poorly understood. To clarify the role of LTβR in cancer cells, LTβR was depleted in melanoma cells using siRNA or the CRISPR/Cas9 system. We observed larger, flattened cells with increased levels of p21 and p53, which are hallmarks of cellular senescence. Conversely, no prominent morphological changes were observed in LTβR-overexpressing cells, but Dox-treated LTβR-overexpressing cells displayed a significant increase in cell number and reduced levels of p53 and p21. Given that p53 mRNA levels remained unchanged while p21 mRNA levels increased, we investigated MDM2 and MDMX, which are known to regulate p53 degradation.

Unlike MDMX, MDM2 has a nuclear localization signal (NLS), and MDM2 is known to auto-ubiquitinate, but under conditions where MDMX binds to MDM2, MDM2 promotes the degradation of MDMX rather than itself [50]. Recent studies linking MDMX to patient survival [51] imply that therapeutic targeting of MDMX may offer a promising strategy for treating cancers in which MDMX modulates p53 activity [24]. We observed that LTβR expression affects p53 degradation and MDMX stability. We hypothesized that LTβR stabilizes MDMX by inhibiting its interaction with MDM2, thereby preventing MDMX nuclear translocation and subsequent degradation, and this regulation of MDMX in the cytoplasm by LTβR may contribute to p53-mediated senescence. In vivo experiments confirmed that LTβR expression affects tumor growth in WT mice, and LTβR knockout tumors exhibited enhanced sensitivity to nutlin-3a, a drug that inhibits MDM2-mediated p53 degradation.

Interestingly, LTβR overexpression induces its self-oligomerization, which leads to its translocation into cells and activation of non-canonical NF-κB signaling [41–44]. This suggests that LTβR-mediated NF-κB signaling can be modulated not only by its ligand binding but also by its own expression. Overexpressing the cytosolic domain of LTβR in HeLa cells, which includes the self-association domain of LTβR, induced cell death [41]. Additionally, LTβR agonist antibodies effectively inhibited tumor growth in colon cancer [10, 17]. Consistent with prior findings, these results suggest LTβR involvement in apoptotic signaling. However, most of the studies indicated that stimulating or overexpressing LTβR induced apoptosis in cells with specific p53 mutations, such as R273H, in the DNA binding domain. For those cells without the p53 mutation, stimulating LTβR has less effect. In this study, we found that knocking down LTβR in HT-29 cells, which also carry the p53 R273H mutation, did not lead to a senescence phenotype, and this suggests that there may be two distinct strategies to target LTβR: blocking it or stimulating it, depending on the p53 status of the cells. We observed that treatment with LIGHT had a paradoxical effect on LTβR, resulting in the downregulation of both p53 and LTβR, while upregulating p21. This study is limited by the absence of experiments involving other ligands and a lack of deeper investigation into the underlying molecular pathways. Therefore, future research should focus on elucidating the molecular interplay between LTβR, NF-κB signaling, and p53 in various cancer types and exploring combination therapies to exploit LTβR–p53 dynamics for improved cancer treatment outcomes. Moreover, studies on inhibitors of LTβR and MDMX, and identifying the precise interaction sites of these molecules are needed to modulate the LTβR–MDMX–p53–p21 axis.

Although we also observed a senescence-like phenotype in other cell lines, we primarily focused on melanoma cell lines to propose a potential cancer-targeting strategy that does not account for immune cell interactions. Additionally, we did not explore gene-targeting strategies in vivo, which may further broaden the therapeutic applicability. Notably, under hypoxic conditions, CREB1 binds to LTβR promoter and regulates its expression [52]. Although LTβR knockdown induces senescence in normal cells such as IMR90, cancer cells are typically exposed to hypoxic environments—where p53 and MDM2 levels are low and LTβR is elevated. In this context, suppressing LTβR levels can serve as a viable strategy. This mechanism may also explain the drug-resistant phenotype observed in patients, further highlighting the potential of targeting LTβR in hypoxic tumors.

In conclusion, our results suggest a potential role for LTβR in regulating p53 by modulating the stability of MDMX, providing insight into its cancer therapeutic strategies.

## Materials and methods

### Cell culture

Human melanoma A375, mouse melanoma B16F10, and human lung fibroblast IMR90 cell lines were cultured in Dulbecco’s Modified Eagle Medium (DMEM, Welgene, Gyeongsan, South Korea). Mouse macrophage J774, human non-small cell lung carcinoma A549, and human colorectal adenocarcinoma HT-29 cell lines were cultured in Roswell Park Memorial Institute (RPMI) medium containing L-glutamine (Welgene). All media were supplemented with 10% heat-inactivated fetal bovine serum (FBS, Corning, Corning, NY, USA). Cells were incubated at 37 °C and 5% CO_2_. Cell lines were purchased from American Type Culture Collection (ATCC) and were confirmed to be free of mycoplasma contamination. For live cell counting, 0.4% trypan blue solution (Gibco, Thermo Fisher Scientific, Waltham, MA, USA) was added to the cell suspension and incubated for 5 min at room temperature (RT). Viable cells, identified as those without staining, were counted using a hemocytometer under a light microscope. Cellular morphological changes were photographed using an inverted phase-contrast microscope.

### Generation of knockout cells

LTβR KO cells were generated using CRISPR/Cas9 KO plasmid system (sc-421483-NIC, Santa Cruz Biotechnology, Dallas, TX, USA) according to the manufacturer’s instructions. After 48 h, transfected cells were selected using 2 μg/ml of puromycin (Sigma-Aldrich, St. Louis, MO, USA). GFP-positive cells were then sorted using BD FACS Aria III (BD Biosciences, Franklin Lakes, NJ, USA) and underwent a second round of puromycin selection. To generate monoclonal populations, transfected cells were diluted to 0.5 cells per well and seeded into 96-well plates. Single colonies were expanded in larger culture vessels, and successful LTβR knockout was confirmed using western blotting.

### Transfection

Human LTβR (HG10581-NF) and mouse LTβR (MG57382-NM) plasmids were obtained from Sino Biological (Wayne, PA, USA). The LTβR-∆ECD plasmid was synthesized and cloned into the same expression vector as full-form LTβR construct in our laboratory. siRNA duplexes against human and mouse LTβR, human MDMX, and nonspecific control siRNA were purchased from Bioneer Inc. (Daejeon, South Korea). Plasmids and siRNA transfections were performed using Lipofectamine 2000 (Invitrogen, Waltham, MA, USA) and RNAiMAX (Invitrogen), respectively, following the supplier’s protocol. After 48 h, cells were subjected to cell counting, SA-β-Gal staining, western blot analysis, real-time PCR, flow cytometry analysis, and immunocytochemistry analysis. For additional experiments, cells were treated with 100 ng/ml doxorubicin (Dox, Cell Signaling Technologies, Danvers, MA, USA), 200 ng/ml recombinant human LIGHT (R&D systems, Minneapolis, MN, USA), and 20 μM nutlin-3a (Selleckchem, Houston, TX, USA) for the indicated time. After transfection, cells were treated with MG-132 (474790, Sigma-Aldrich) for 4 h, and cyclohexamide (CHX, C4859, Sigma-Aldrich) for the designated time.

### Western blot analysis

Cells were collected and lysed in RIPA buffer containing protease and phosphatase inhibitors. Nuclear/cytosol fractionation was performed (ab289882, Abcam, Cambridge, UK) according to the manufacturer’s protocol. Protein concentrations were quantified using bicinchoninic acid assay. Equal amounts of total protein were resolved via SDS-PAGE and transferred onto nitrocellulose membranes. The membranes were blocked with 5% skim milk or 5% BSA in 0.1% TBS-T. Proteins were detected using the following specific antibodies: α-LTβR (20331-1-AP, Proteintech, Rosemont, IL, USA or PA5-88290, Invitrogen), α-p53 (10442-1-AP, Proteintech), α-p21 (556431, BD Biosciences), α-MDM2 (ab259265, Abcam), α-MDMX (17914-1-AP, Proteintech), α-IκBα (4812S, Cell Signaling Technology, Danvers, MA, USA), α-p52 (4882, Cell Signaling Technology), α-USP7 (66514-1-Ig, Proteintech), α-TRAF6 (8028S, Cell Signaling Technology), α-lamin B1 (ab16048, Abcam), α-GAPDH (AC002, Abclonal, Wuhan, China), and α-β-actin (sc-47778, Santa Cruz) were used. HRP-conjugated secondary antibodies (Jackson ImmunoResearch, West Grove, PA, USA) were used to detect antigen-antibody complexes, which were further visualized using enhanced chemiluminescent substrate (ECL, GenDEPOT, Barker, TX, USA). The membranes were stripped by submerging them in stripping buffer (Biomax, Rockville, MD, USA) for 20 min under constant shaking at RT.

### Confocal microscopy

Cells were cultured in four-well glass slides (SPL Life Sciences, Pocheon, South Korea) and fixed using 4% paraformaldehyde solution. After permeabilization with 0.1% Triton X-100, cells were stained using the specific antibodies, followed by a fluorochrome-tagged secondary antibody. The following antibodies were used: α-MDMX (17914-1-AP, Proteintech), α-p21 (556431, BD Biosciences), and α-Ki67 (ab15580, Abcam). Slides were mounted using Fluoromount-G™ Mounting Medium, with DAPI (Invitrogen) and observed under confocal microscopy (ZEISS, LSM700, Jena, Germany). To quantify nuclear MDMX fluorescence intensity relative to cytosolic MDMX, we analyzed green fluorescence images using ImageJ. Nuclear regions were identified by overlaying the DAPI-stained image, and these nuclear regions were excluded to isolate the cytosolic signal. The fluorescence intensity was measured separately in the cytoplasmic and nuclear compartments. To normalize the data, the total intensity was divided by the number of nuclei (determined by DAPI-positive cells).

### Real-time PCR

Total RNA was extracted 48 h post-transfection using AccuPrep® Universal RNA Extraction Kit (Bioneer, Daejeon, South Korea) following the manufacturer’s protocol. Oligo (dT) primer was used to generate 2 μg of cDNA in premix (Takara Bio Inc., Shiga, Japan). Real-time PCR was performed using the Power SYBR™ Green PCR Master Mix (Thermo Scientific), as per the manufacturer’s instructions. Relative quantification of mRNA expression levels was calculated using the 2^-ΔΔCt^ method. All real-time PCR reactions were performed in triplicate.

### Immunoprecipitation

Interaction between LTβR and MDMX was confirmed by immunoprecipitation. Cells were lysed using RIPA buffer and centrifuged at 4 °C, 15,000 rpm for 40 min. Dynabeads^TM^ Protein G (Invitrogen) were pre-incubated with ~1–2 μg of MDMX antibody (17914-1-AP, Proteintech), LTβR antibody (16-5671-82, Invitrogen), α-USP7 (66514-1-Ig, Invitrogen), Flag antibody (F7425, Sigma-Aldrich), or IgG (12-371, Sigma-Aldrich) for 1 h at RT. The antibody-coated beads were further incubated with cell lysate. After 3 washes with lysis buffer, the bound proteins were eluted by boiling the beads at 100 °C for 7 min. For immunoprecipitation of ubiquitinated protein, cells were treated with bortezomib (BTZ, 50 nM) for 4 h, and N-ethylmaleimide (NEM, 10 mM) was added in the lysis buffer. After centrifugation of lysate, residual NEM in the supernatants was neutralized by adding dithiothreitol (DTT) to a final concentration of 10 mM, as previously described [53].

### Proximity ligation assay (PLA)

PLA was performed after 48 h of LTβR siRNA or plasmid transfection in both A375 and B16F10 cells. Cells were cultured in 8-well Nunc™ Lab-Tek™ Chamber Slide System (Thermo Scientific) and fixed using 4% paraformaldehyde for 10 min at RT. Following permeabilization with 0.1% Triton X-100, the Duolink® Proximity Ligation Assay (Sigma-Aldrich) was performed according to the manufacturer’s protocol. A375 cells were incubated with antibodies to LTβR (20331-1-AP, Proteintech), MDMX (sc-374147, Santa Cruz), and MDM2 (ab259265, Abcam). While B16F10 cells were incubated with antibodies to LTβR (16-5671-82, Invitrogen) and MDMX (17914-1-AP, Proteintech).

### SA-β-Gal staining

To evaluate cellular SA-β-Gal activity, SA-β-Gal staining was performed on day 2 post-treatment using β-Galactosidase staining solution (pH 6.0, 5 mM potassium ferrocyanide, 5 mM potassium ferricyanide, 40 mM citric acid/sodium phosphate, 150 mM NaCl, 2 mM MgCl_2_, and 1 mg/ml X-Gal) or CellEvent™ Senescence Green Detection Kit (Invitrogen). Cellular morphological changes were photographed using an inverted phase-contrast microscope (Olympus, Tokyo, Japan). For tissue SA-β-Gal staining, excised tumors were washed with PBS and embedded in an O.C.T. Compound (Leica Biosystems, Buffalo Grove, IL, USA). Frozen tissues were sectioned into 20 µm-thick slices using a cryostat (Leica Biosystems). Cryosections were washed with 1x PBS, fixed with 0.2% glutaraldehyde for 10 min, RT, and stained with a β-Galactosidase staining solution. Sections were incubated at 37 °C for 12–16 h. After washing twice with PBS, sections were stained with hematoxylin for nuclear visualization and covered with a cover glass using a Fluoromount-G™ Mounting Medium (Invitrogen). Images were obtained using an inverted phase-contrast microscope [54].

### Mouse experiment

All animal procedures were approved by the Institutional Animal Care and Use Committee (IACUC no. 2023-0146). Briefly, 8-week-old female BALB/c mice were housed in a specific pathogen-free facility and were used for allograft tumor experiments. Mice were randomly assigned, but the experiments were not blinded. To generate tumors, 1 × 10^6^ B16F10^WT^ and B16F10^LTβR-KO^ cells were suspended in 100 μl PBS, injected into the dorsal subcutaneous area of mice, and successfully formed tumor masses after implantation. Mice were administered a single intraperitoneal injection of doxorubicin (4 mg/kg body weight) or nutlin-3a (20 mg/kg body weight) after tumor formation, and tumors were collected after 7 days. Tumor tissues were fixed overnight in 4% formalin and embedded in paraffin. These sections were prepared for immunohistochemistry to detect p21 and MDMX. Subsequently, the slices were counterstained with DAPI. Images were captured from randomly selected areas of each tumor section, following standard protocols.

### Statistical analysis

Statistical analysis and data visualization were conducted using GraphPad Prism 10 software (GraphPad Software, San Diego, CA, USA). For comparisons between two groups, statistical significance was determined by unpaired *t*-test or multiple unpaired *t*-tests with false discovery rate (FDR) correction, using a threshold of FDR < 0.01. For comparisons involving two independent variables, two-way ANOVA was performed, followed by uncorrected Fisher’s least significant difference (LSD) test, Tukey’s multiple comparison test, or Šidák’s multiple comparison test. Each experiment was independently performed at least three times, with similar results.

## Supplementary information

Supplementary Material (Supplementary figures 1-7)

Supplementary Material (Western blot original data)

## Data Availability

Original data are available upon request. The full length, uncropped original western blots are shown in the ‘Supplementary Material’.
