# Supplementary material for: Reduction of lymphotoxin beta receptor induces cellular senescence via the MDMX-p53 pathway
Source: Cell Death Discov. 2025 Aug 29;11:416. doi: 10.1038/s41420-025-02708-1 (PMC12397326; doi:10.1038/s41420-025-02708-1)
Supplement: Supplementary file 1 — Supplementary Material (Supplementary figures 1-7) [file 41420_2025_2708_MOESM1_ESM.docx]

Supplementary information

**Reduction of lymphotoxin beta receptor induces cellular senescence via the MDMX-p53 pathway**

So Young Kim^1,3^, Bin Lee^1,2^, Je-Jung Lee^1,2^, Man Sup Kwak^1,2^, Woo Joong Rhee^1,2^, In Ho Park^2,4^, Jeon-Soo Shin^1–3, #^

^1^Department of Microbiology, Yonsei University College of Medicine, Seoul, South Korea.

^2^Institute for Immunology and Immunological Diseases, Yonsei University College of Medicine, Seoul, South Korea.

^3^Brain Korea 21 FOUR Project for Medical Science, Yonsei University College of Medicine, Seoul, South Korea.

^4^Department of Biomedical Sciences, Yonsei University College of Medicine, Seoul, South Korea.

^#^ Corresponding Author:

Jeon-Soo Shin

Department of Microbiology, Yonsei University College of Medicine, 50-1 Yonsei-ro, Seodaemun-gu, Seoul 03722, South Korea

Tel: +82-2-2228-1816

Fax: +82-2-392-7088

E-mail: jsshin6203@yuhs.ac

**Running Title:** Lymphotoxin β receptor prevents senescence via inhibition of MDMX nuclear translocation.

**Supplementary methods**

**Cell cycle analysis**

Cells were detached from the culture plate into single-cell suspension using 0.25% Trypsin-EDTA (Gibco, Thermo Fisher Scientific) and washed twice with PBS. Cells were fixed in ice-cold 70% ethanol for 2 h at 4°C, followed by incubation with 100 μg/ml of RNase (QIAGEN, Hilden, Germany) at 37°C for 30 min. After thorough washing, cells were stained with 1 μg/ml propidium iodide (Invitrogen) and analyzed for cell cycle distribution using FACSVerse II flow cytometer (BD Biosciences).

**Supplementary Figures**


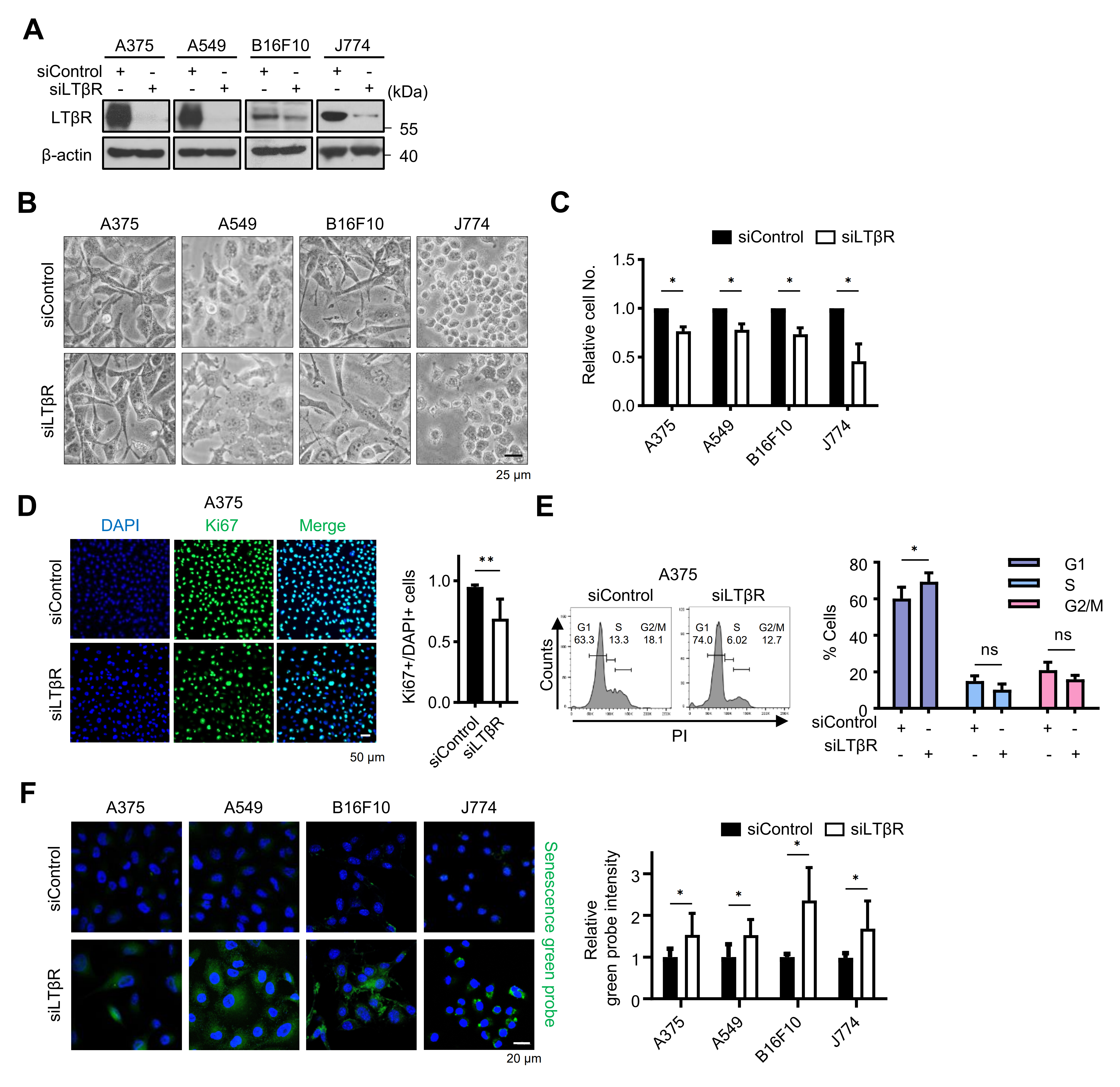


**Supplementary Figure 1. LTβR depletion induces senescence in various cells.** A375 (human melanoma cell line), A549 (human non-small cell lung carcinoma cell line), B16F10 (mouse melanoma cell line), and J774 (mouse macrophage cell line) cells were transfected with 100 nM of siControl (control siRNA) or siLTβR (LTβR siRNA). Cells were collected 48 hours after siRNA transfection for (A) western blot analysis of the indicated proteins. Control and LTβR knockdown A375, A549, B16F10, and J774 cells were subjected to phase-contrast microscopy for (B) morphological changes and (C) cell counting to evaluate relative cell number. (D) Confocal microscopy was used to detect Ki67 expression in LTβR knockdown A375 cells, with relative fluorescence intensity quantified using ImageJ. (E) Cell cycle distribution was analyzed using propidium iodide (PI) staining. (F) Confocal imaging with a senescence green probe was performed to evaluate senescence. Fluorescence intensity for relative senescence green probe in LTβR knockdown cells were quantified using ImageJ software. β-actin was used as a loading control. Graphical data are presented as means ± SD from three independent experiments (n = 3). Asterisks indicate that comparisons were statistically significant following FDR correction (C, F). ** *p* <0.01, using an unpaired Student's t-test (D). **p* <0.05 using Šidák’s multiple comparison test (E).


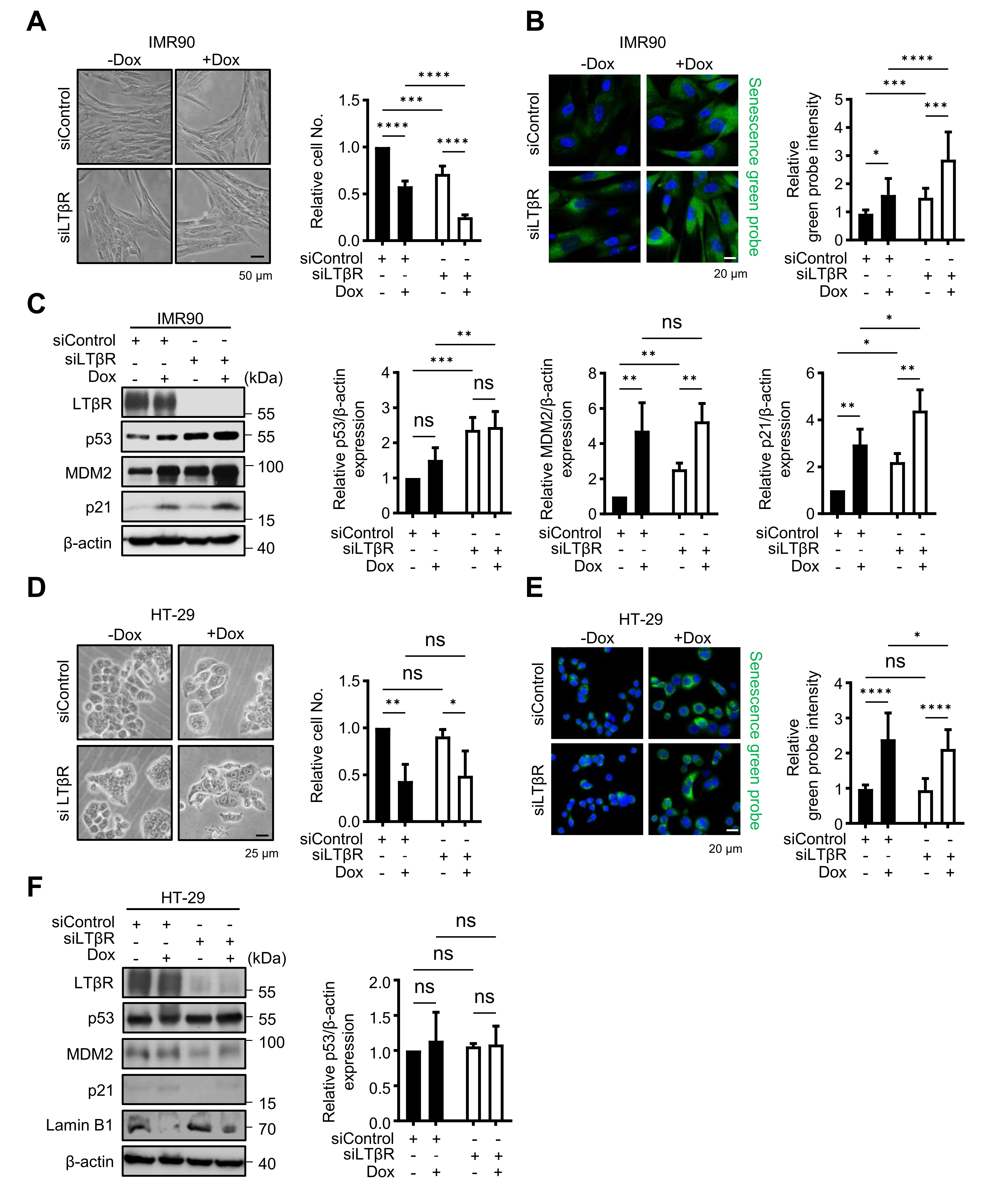


**Supplementary Figure 2. LTβR knockdown induces senescence in normal cells but not in cancer cells with mutated p53.** IMR90 (normal human lung fibroblast) cells were transfected with 100 nM of siControl or siLTβR followed by 100 ng/ml Dox treatment for 48 h. (A) Morphological changes and relative cell number were assessed. (B) Confocal images of a senescence green probe. (C) Western blot images of the indicated proteins and relative band intensities of p53, MDM2, and p21. (D, E) HT-29 (colorectal adenocarcinoma) cells were transfected with 100 nM of siControl or siLTβR and treated with 100 ng/ml Dox for 48 h. Cells were collected for (D) morphological changes and relative cell number, (E) senescence green probe confocal images, and (F) western blot images of the indicated proteins and p53 quantification. Bands and fluorescence intensity for relative senescence green probe were quantified using ImageJ software. Data are represented as mean ± SD from three independent experiments (n = 3). **p* <0.05, ***p* <0.01, ****p* <0.001, *****p* <0.0001, using Fisher’s LSD post hoc test. n.s, not significant.


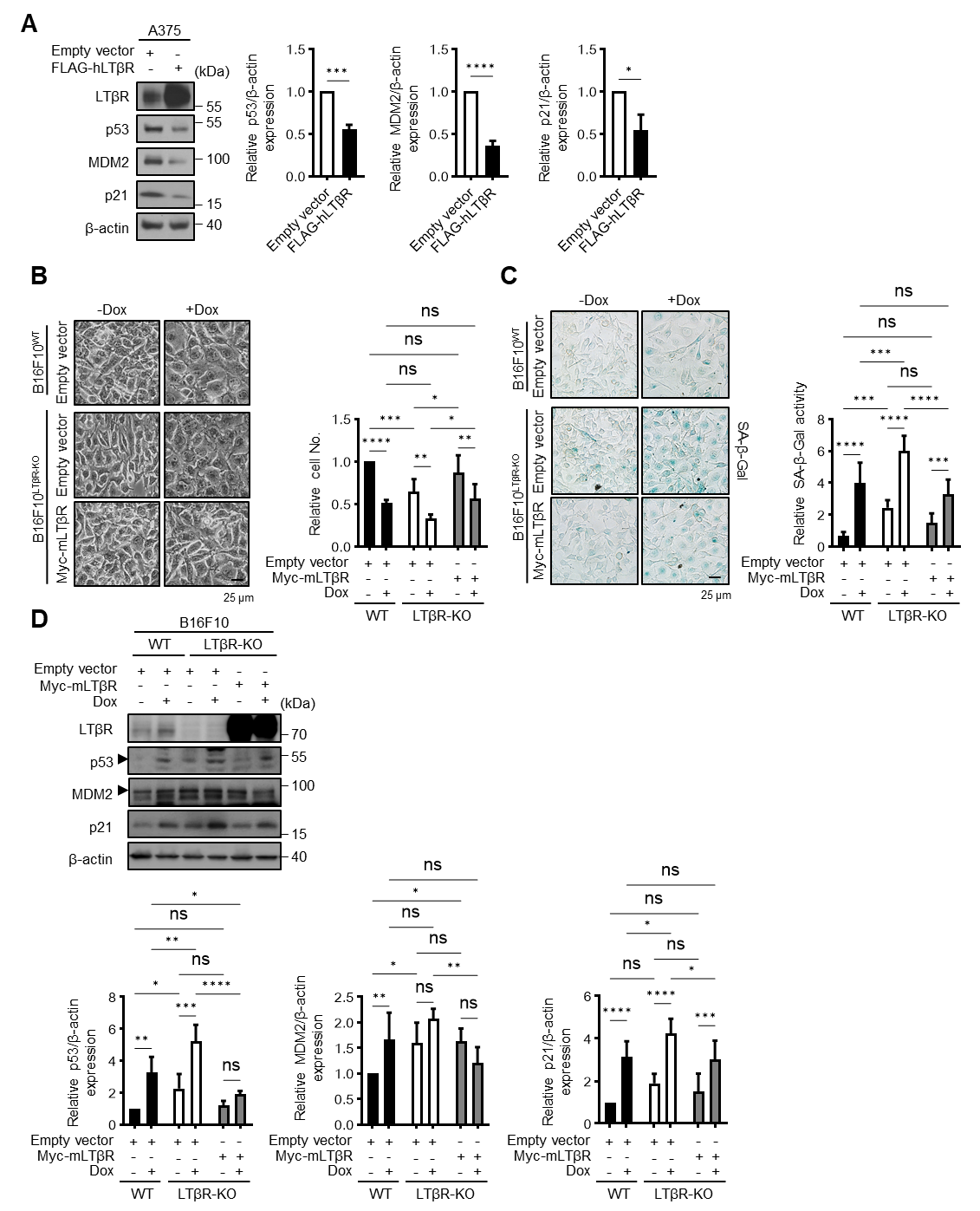


**Supplementary Figure 3. LTβR restoration alleviates senescence.** (A) A375 cells were transfected with LTβR plasmid. Western blot images of the indicated proteins of A375 cells and relative band intensities of p53, MDM2, and p21. B16F10^WT^ and B16F10^LTβR-KO^ cells were transfected with LTβR plasmid followed by 100 ng/ml Dox treatment for 48 h. Cells were collected for (B) morphological change and relative cell number, (C) phase-contrast microscope imaging of SA-β-Gal staining, and (D) western blot imaging of the indicated proteins. Relative SA-β-Gal activity, p53, MDM2, and p21 protein levels were quantified using ImageJ software. Graphical data are represented as mean ± SD from three independent experiments (n = 3). **p* <0.05, *** *p* <0.001, **** *p* < 0.0001, using an unpaired Student's t-test (A). **p* <0.05, ***p* <0.01, ****p* <0.001, *****p* <0.0001, using Tukey’s honestly significant difference post hoc test for multiple comparisons. n.s, not significant.

**
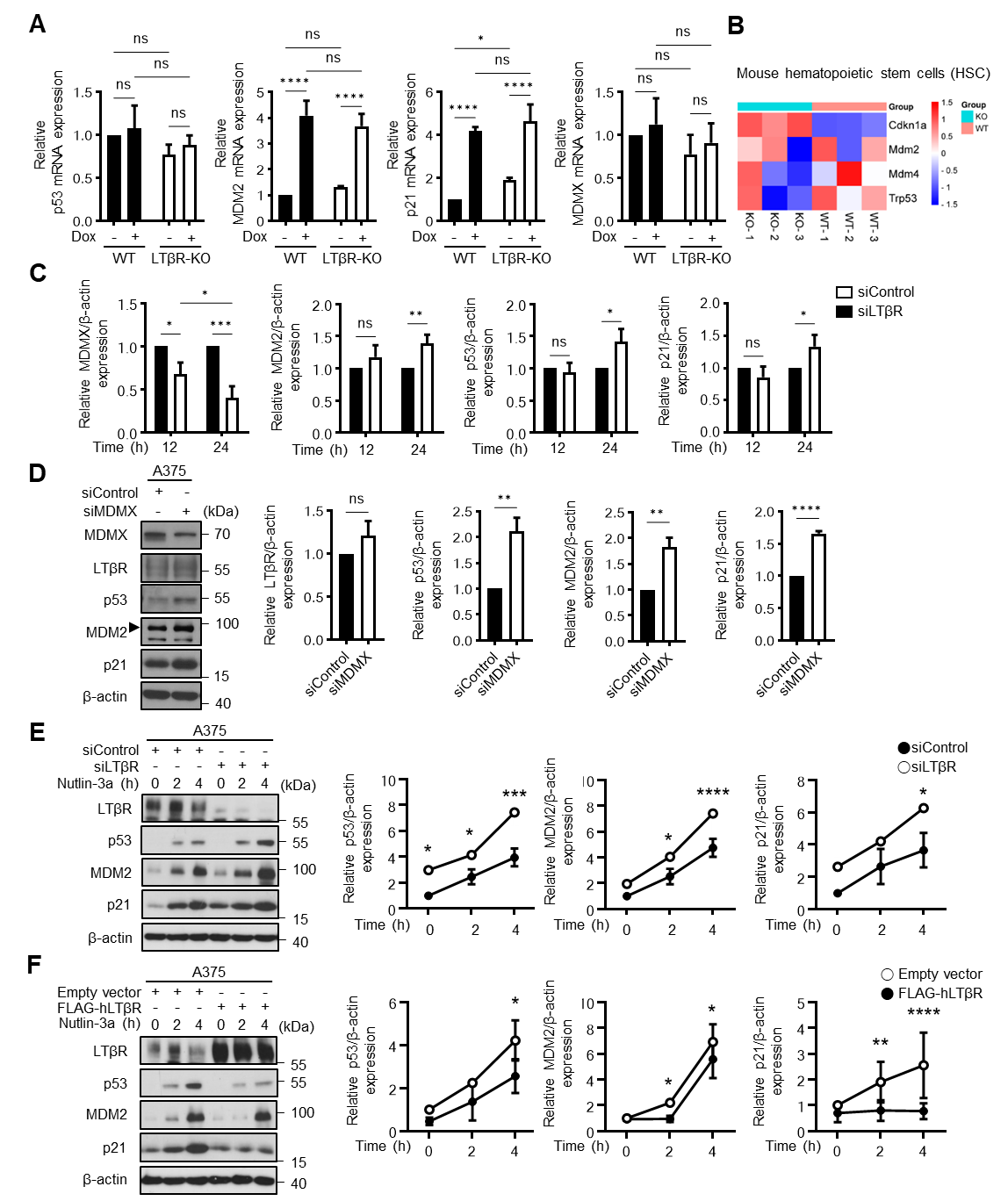
**

**Supplementary Figure 4. LTβR and MDMX regulate p53 protein levels.** (A) Relative mRNA expression levels of *tp53 (*p53*), cdkn1a (*p21*), mdm2, mdm4 (*MDMX*)* genes in B16F10^WT^ and B16F10^LTβR-KO^ cells determined by real-time PCR 48 h after Dox treatment (n = 4). (B) Heat map image from RNA-seq (GSE141206) of LTβR knock-out mouse hematopoietic stem cells and wild-type HSC. (C) Quantification of the indicated proteins shown in Figure 3D. (D) A375 cells were subjected to western blotting after 48 h of siControl or siMDMX (MDMX siRNA) transfection. (E, F) LTβR knockdown and LTβR-overexpressing A375 cells were treated with 20 μM of nutlin-3a for the indicated durations and subjected to western blotting. Graphical data are represented as mean ± SD from three independent experiments (n = 3). **p* <0.05, ****p* <0.001, *****p* <0.0001, using Šidák’s multiple comparison test or Fisher’s LSD post hoc test for multiple comparisons. n.s or absence of a symbol indicates not significant (A, C, E, F). ***p* <0.01, *****p* <0.0001, using an unpaired Student's t-test. n.s, not significant (D).

**Supplementary Figure 5. The extracellular domain of LTβR is not required to prevent senescence.** A375 cells were transfected with extracellular domain (ECD)-deleted LTβR plasmid (FLAG-hLTβR-ΔECD) and treated with 100 ng/ml of Dox for 48 h. Cells were collected and analyzed for (A) morphological changes and relative cell numbers, (B) confocal imaging using a senescence green probe, and (C) protein expression using western blot. Relative fluorescence intensities of the senescence green probe and band intensities of p53, MDM2, and p21 were quantified using ImageJ software. Graphical data are presented as means ± SD from three independent experiments (n = 3). **p* <0.05, ***p* <0.01, ****p* <0.001, *****p* <0.0001, using Fisher’s LSD post hoc test. n.s, not significant.


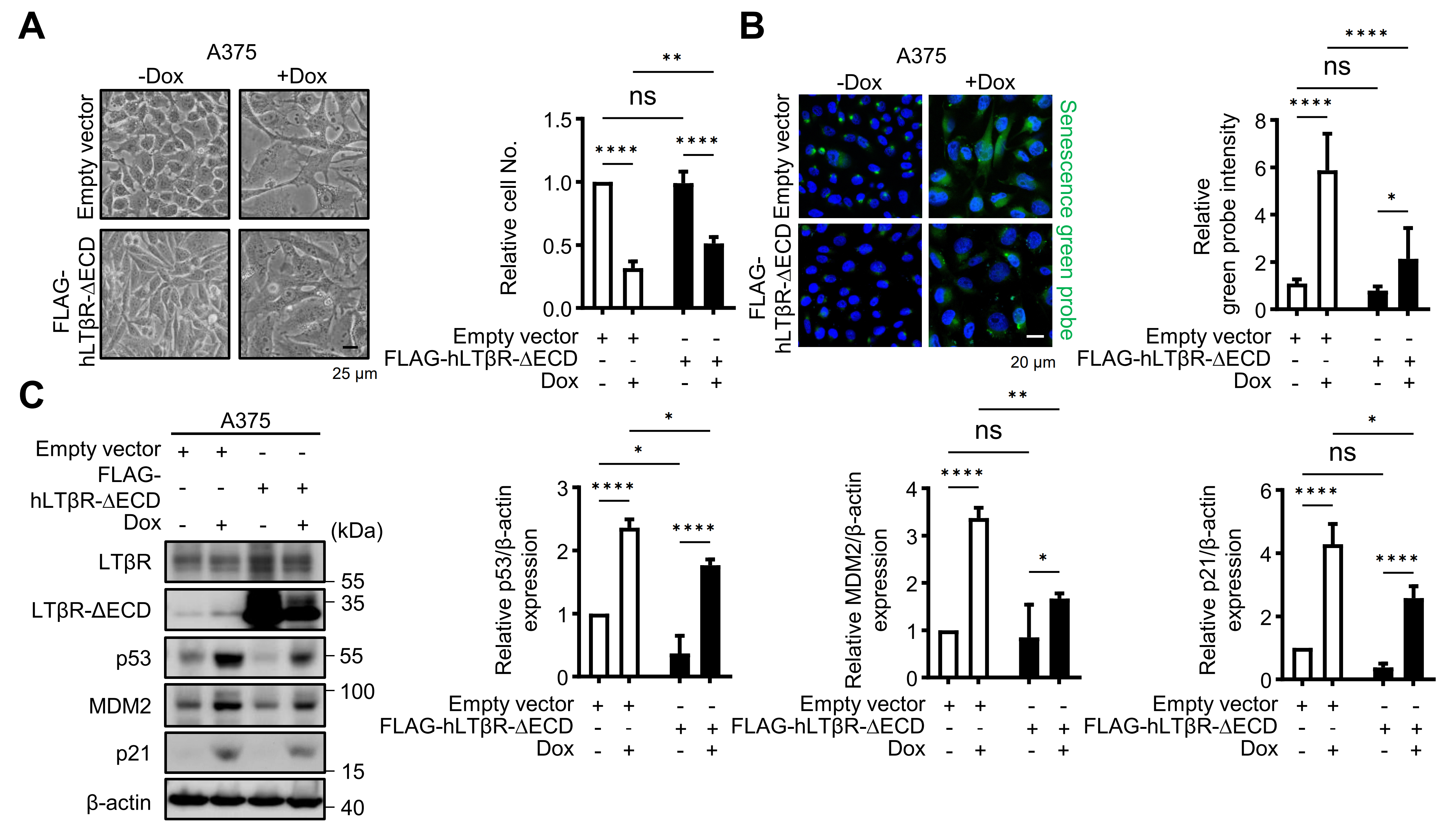


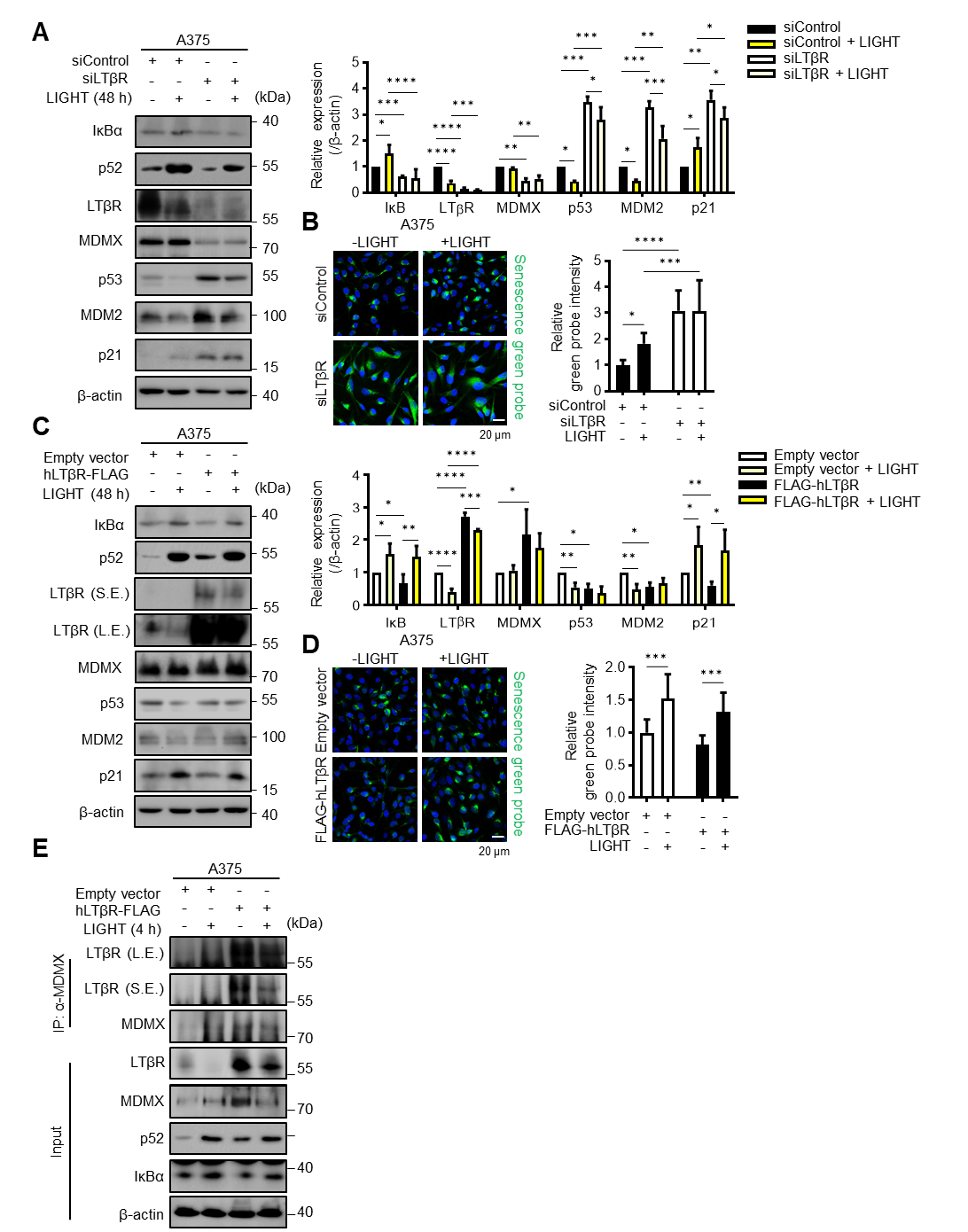


**Supplementary Figure 6. The LTβR ligand is dispensable for the induction of p53-mediated senescence.** A375 cells were transfected with siLTβR (A, B) or LTβR plasmid (C, D) and treated with 200 ng/ml of LIGHT for 48 h. Cells were collected and analyzed for protein expression by western blot (A, C) and SA-β-Gal activity was assessed by confocal imaging using a senescence green probe (B, D). Relative fluorescence intensities of the probe and band intensities from western blots were quantified using ImageJ software. (E) A375 cells were transfected with LTβR plasmid for 48 h and then treated with 200 ng/ml of LIGHT for 4 h. To examine whether the interaction between LTβR and MDMX is affected by LIGHT treatment, cells were collected and subjected to immunoprecipitation using MDMX antibodies, followed by western blotting for the indicated proteins. Graphical data are presented as means ± SD from three independent experiments (n = 3). **p* <0.05, ***p* <0.01, ****p* <0.001, *****p* <0.0001 using Fisher’s LSD post hoc test for multiple comparisons. n.s or absence of a symbol indicates not significant.


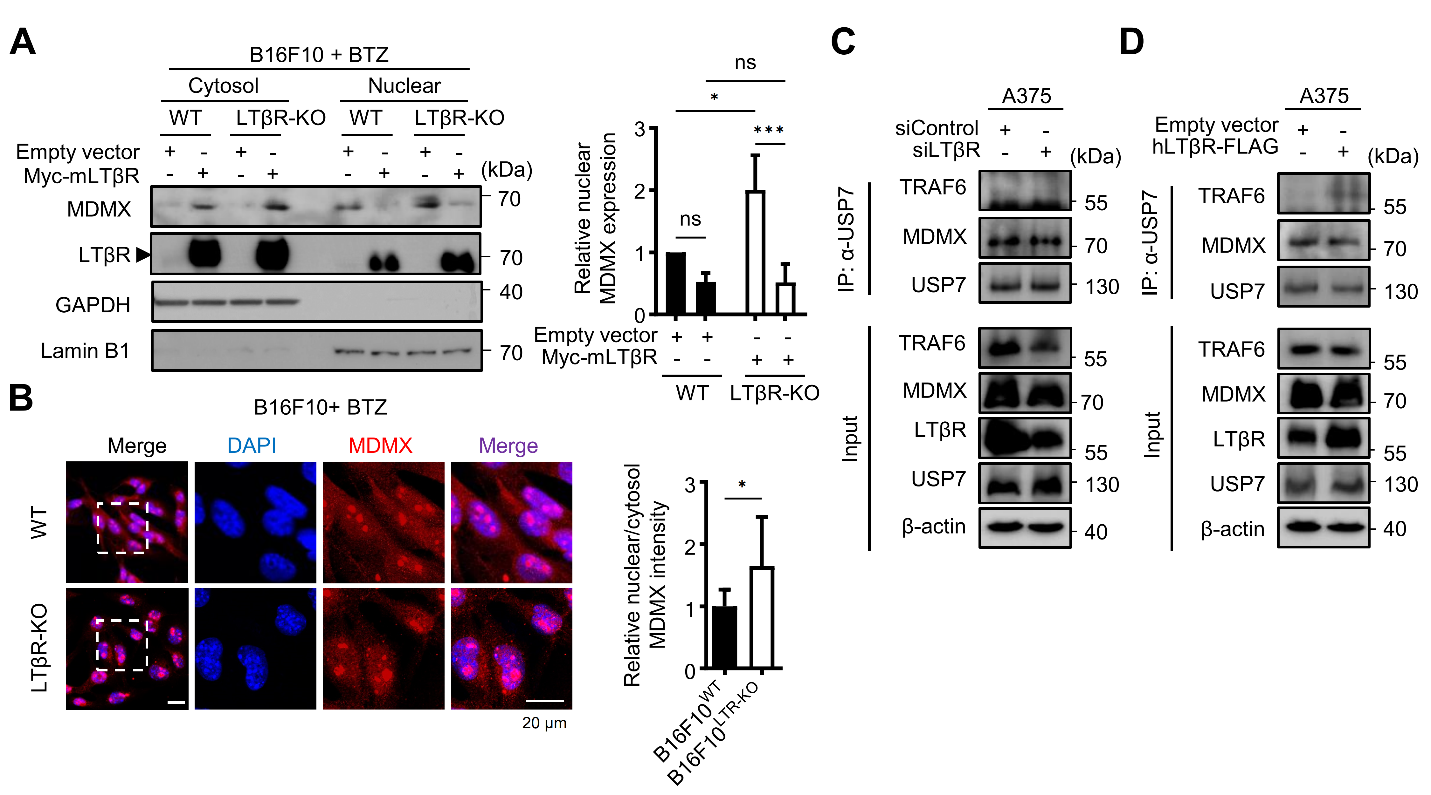


**Supplementary Figure 7. LTβR restricts MDMX nuclear accumulation.** (A) B16F10^WT^ and B16F10^LTβR-KO^ cells were transfected with LTβR plasmid and treated with 80 nM BTZ followed by nuclear fractionation for western blotting. Relative nuclear MDMX band intensities were quantified using ImageJ software, with GAPDH (cytosol) and Lamin B1 (nuclear) serving as loading controls. (B) Confocal microscopy of 80 nM BTZ-treated B16F10^WT^ and B16F10^LTβR-KO^ cells, with relative fluorescence intensity of nuclear MDMX compared to cytosolic MDMX quantified using ImageJ. (C) A375 cells were transfected with 100 nM of siControl (control siRNA) or siLTβR (LTβR siRNA). Cells were collected 48 h after siRNA transfection for immunoprecipitation using USP7 antibodies and western blot of indicated proteins. (D) A375 cells were transfected with LTβR plasmid for 48 h and subjected to immunoprecipitation using USP7 antibodies and western blot of indicated proteins. Graphical data are presented as means ± SD from three independent experiments (n = 3). **p* <0.05, ****p* <0.001, using Fisher’s LSD post hoc test for multiple comparisons (A) or an unpaired Student's t-test (B). n.s, not significant.
