# Supplementary figures and images for: Reduction of lymphotoxin beta receptor induces cellular senescence via the MDMX-p53 pathway
Source: Cell Death Discov. 2025 Aug 29;11:416. doi: 10.1038/s41420-025-02708-1 (PMC12397326; doi:10.1038/s41420-025-02708-1)

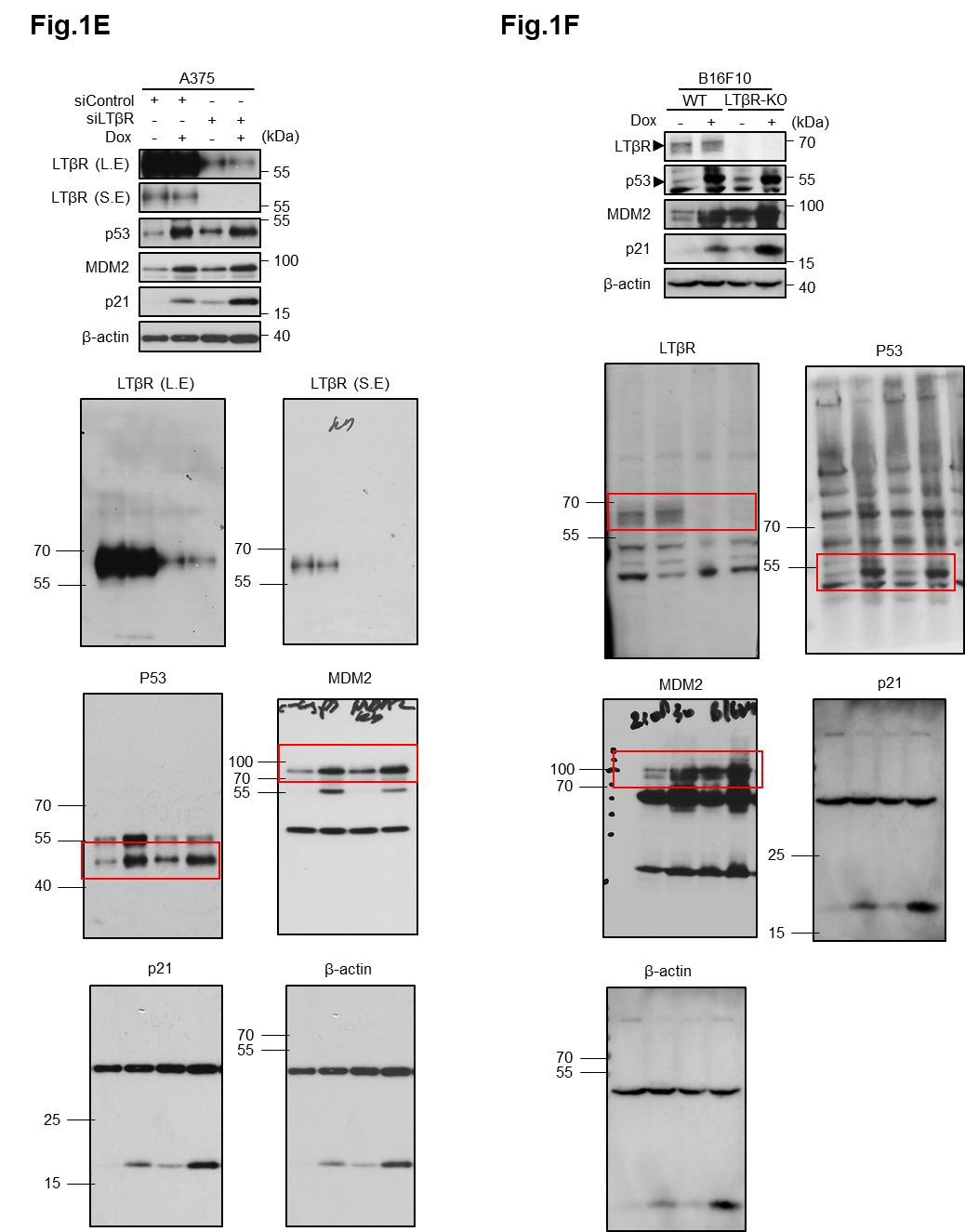


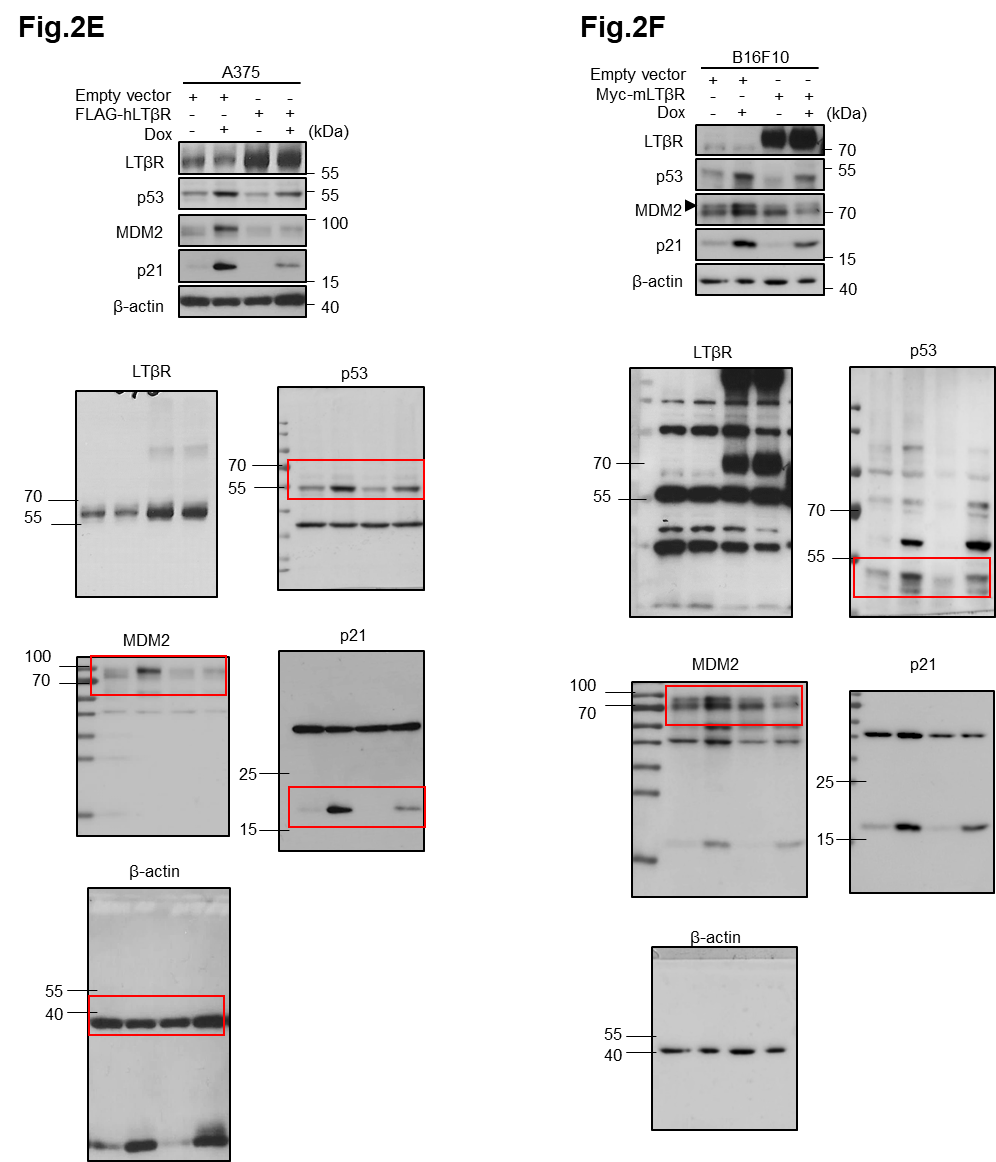


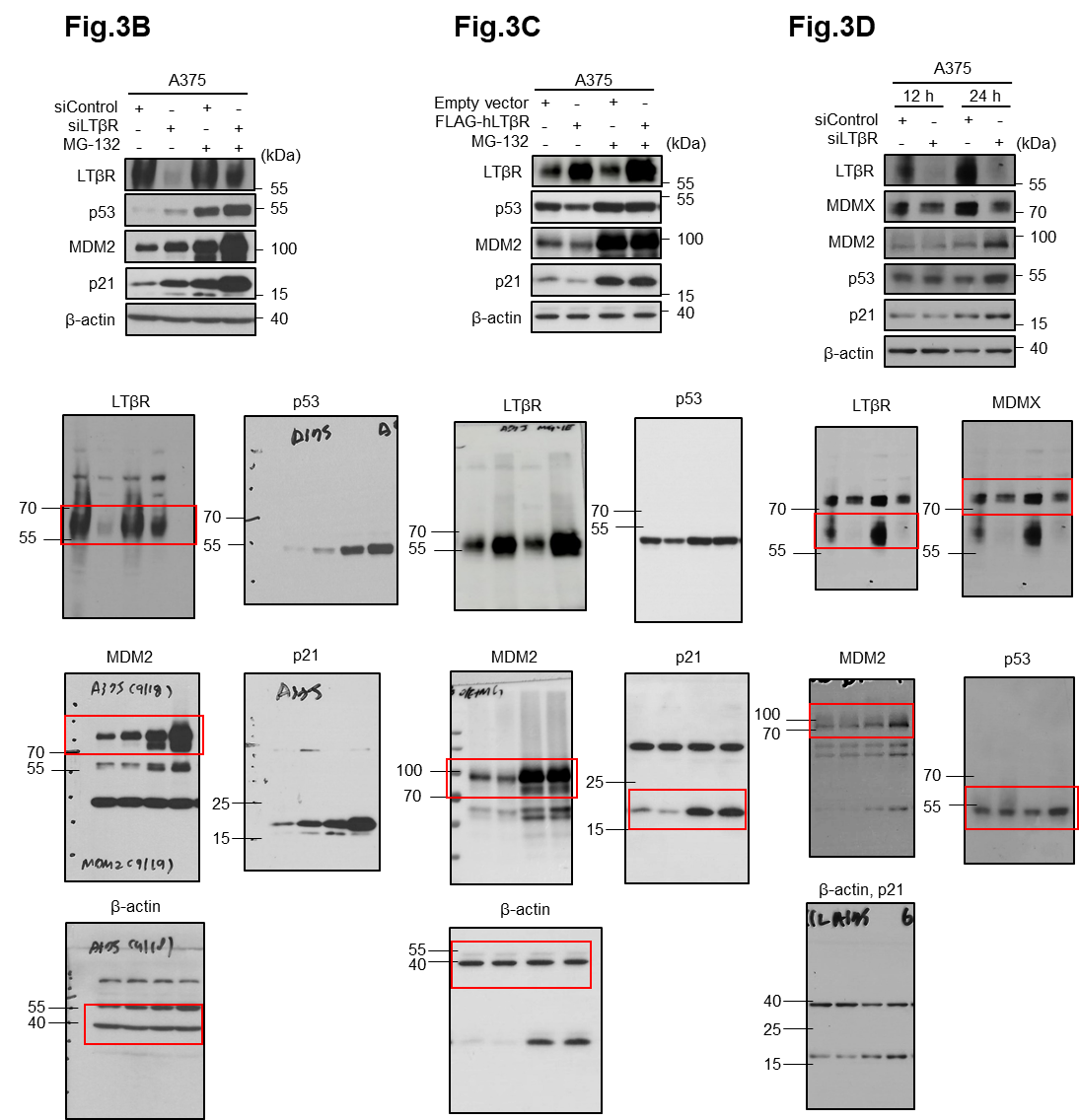


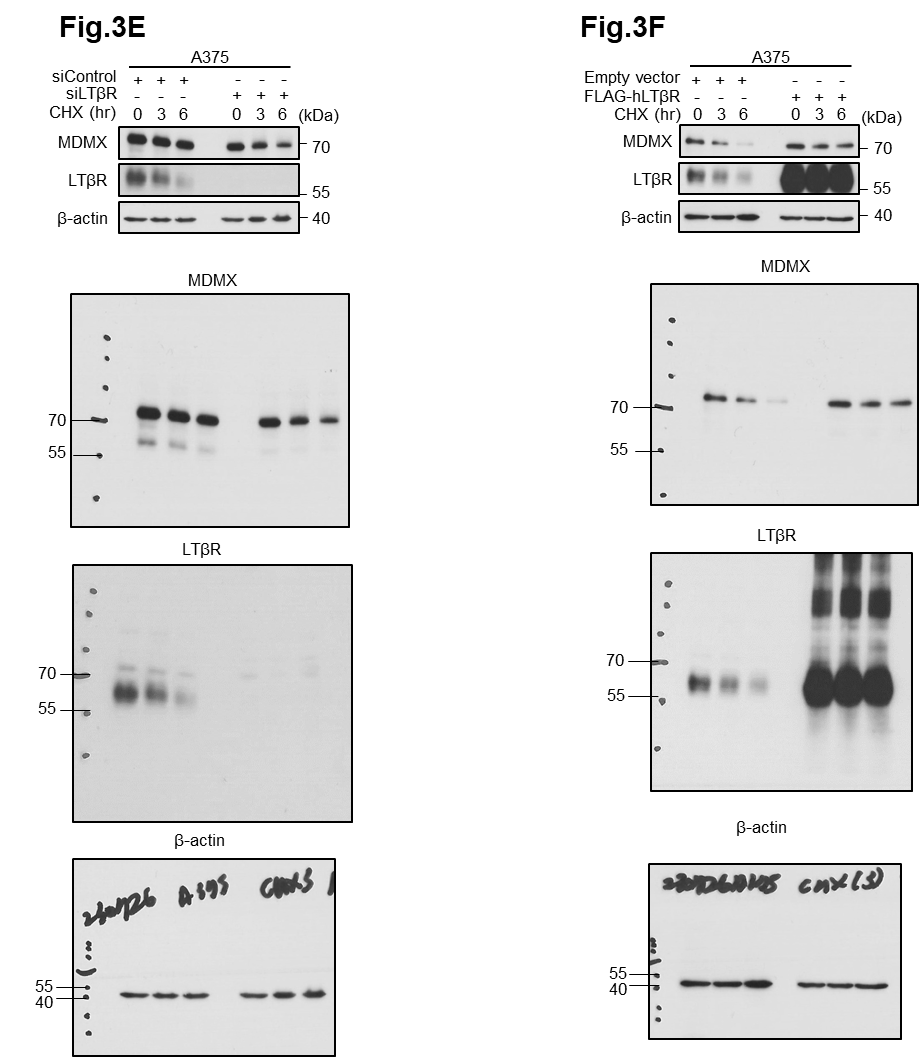


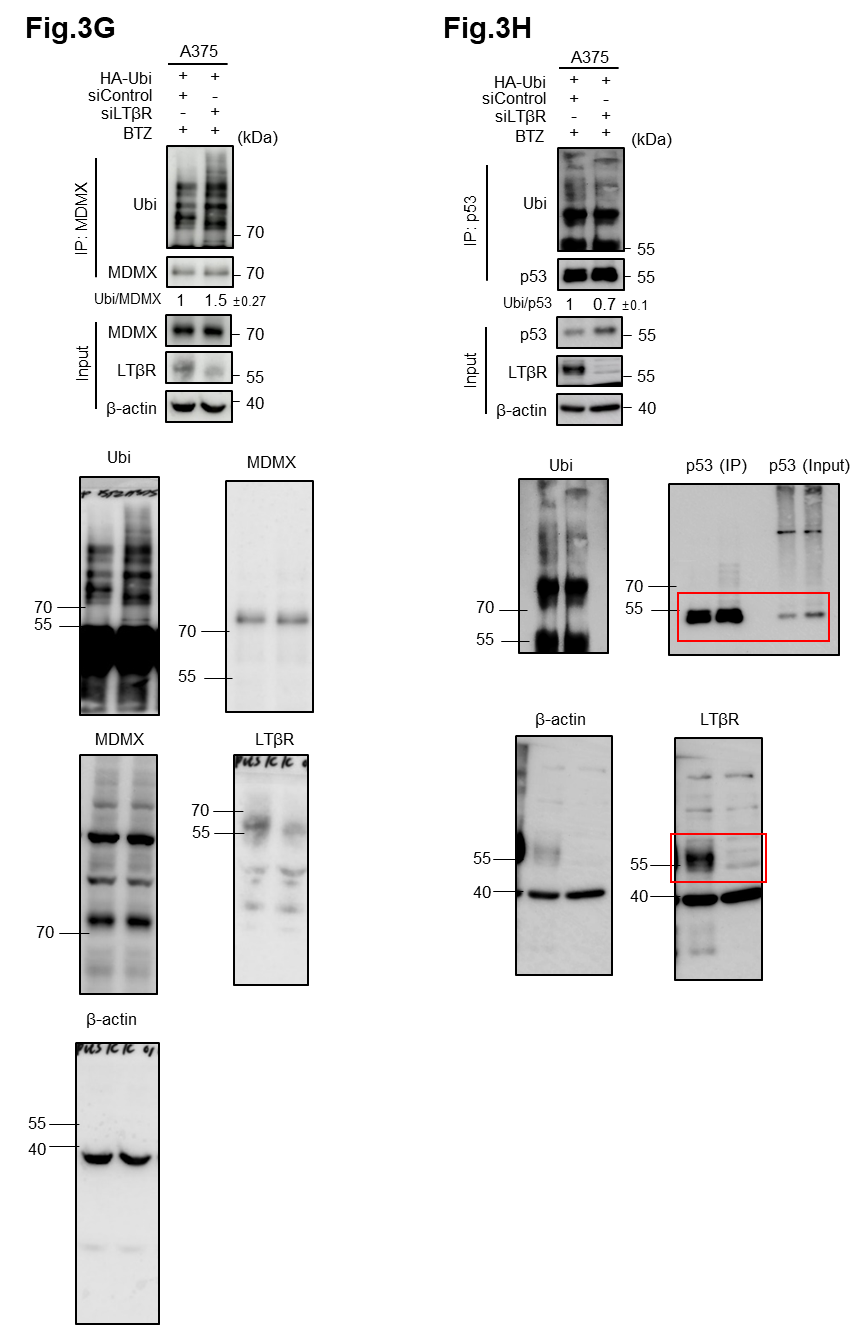


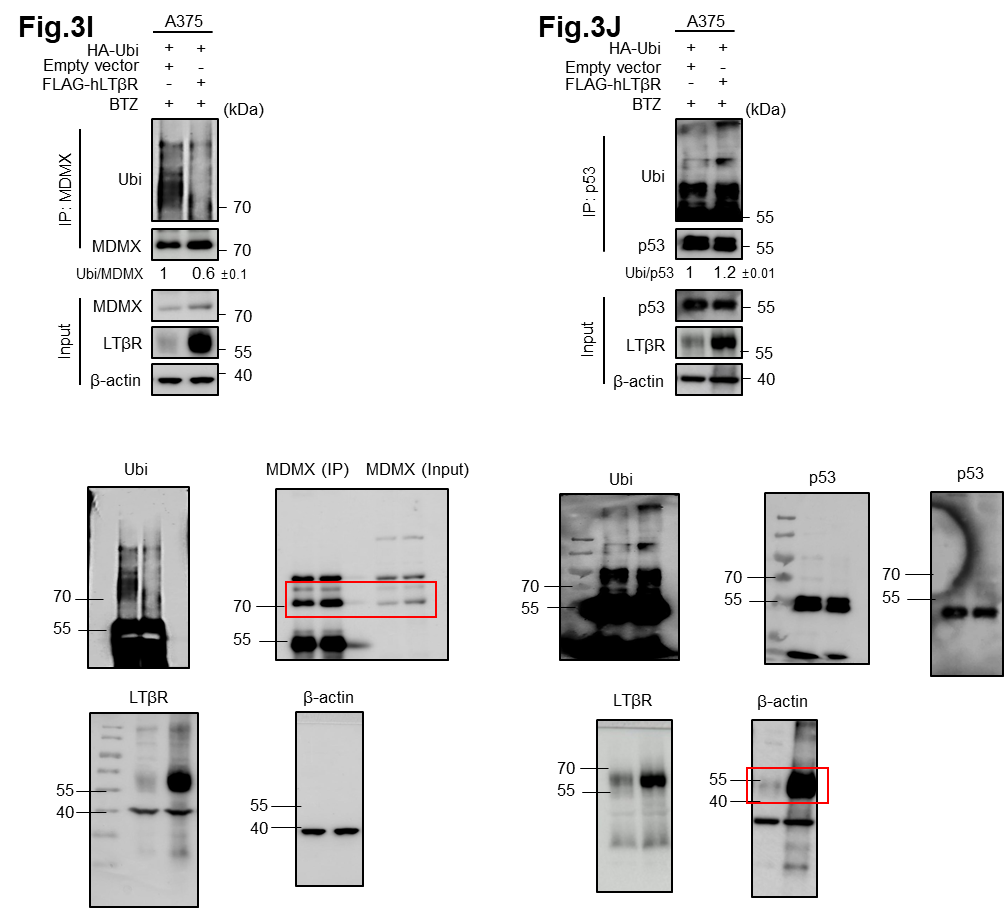


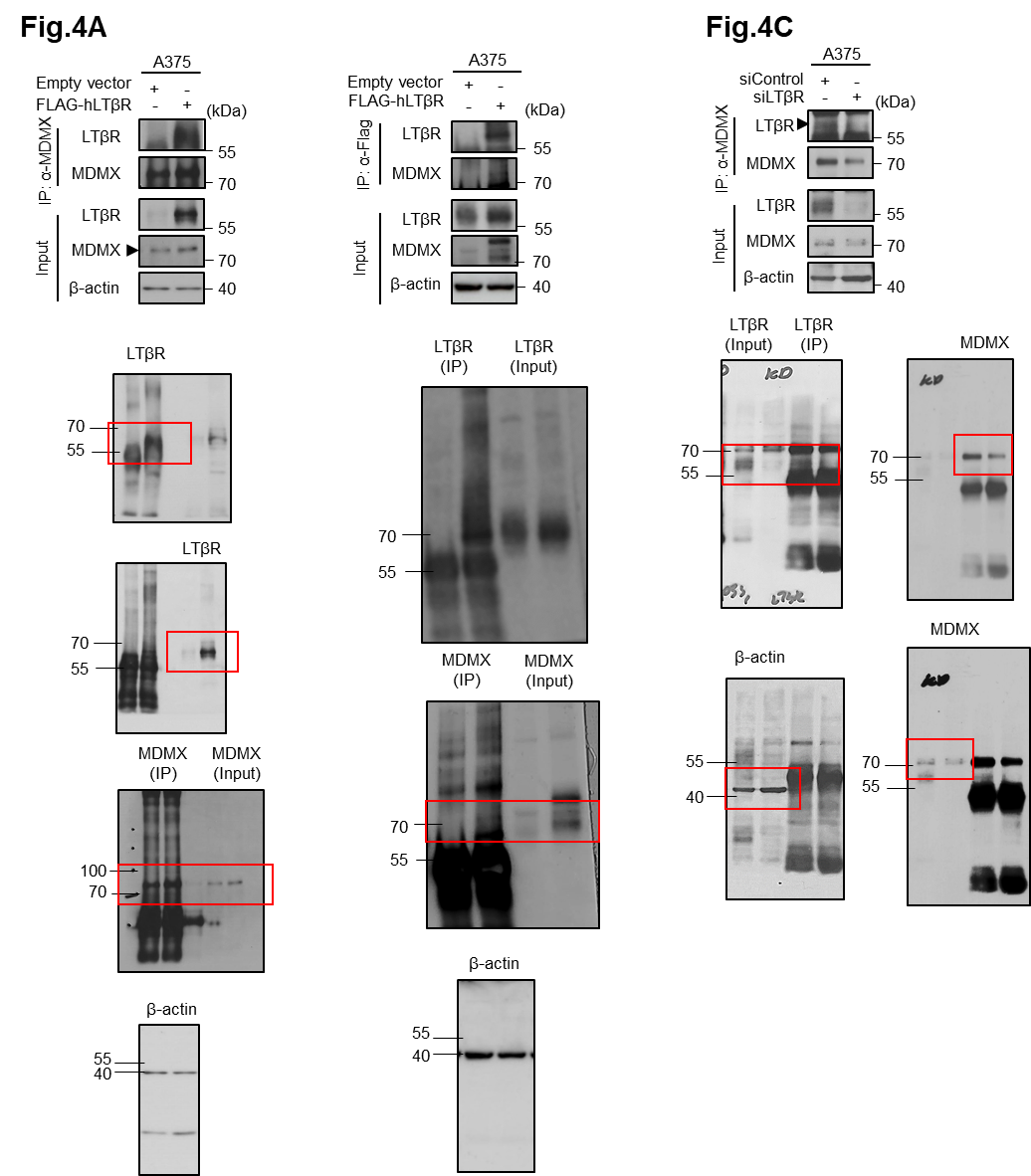


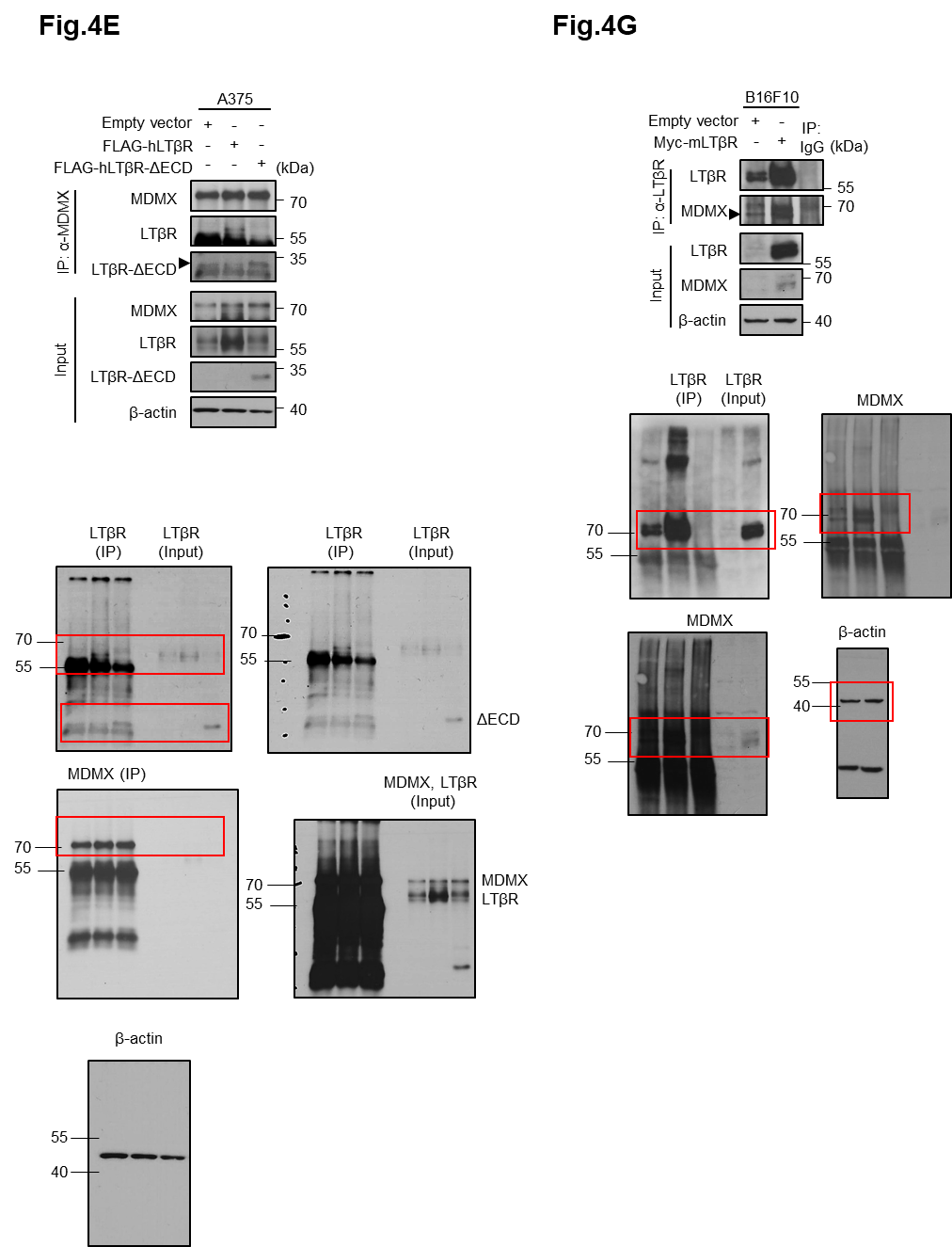


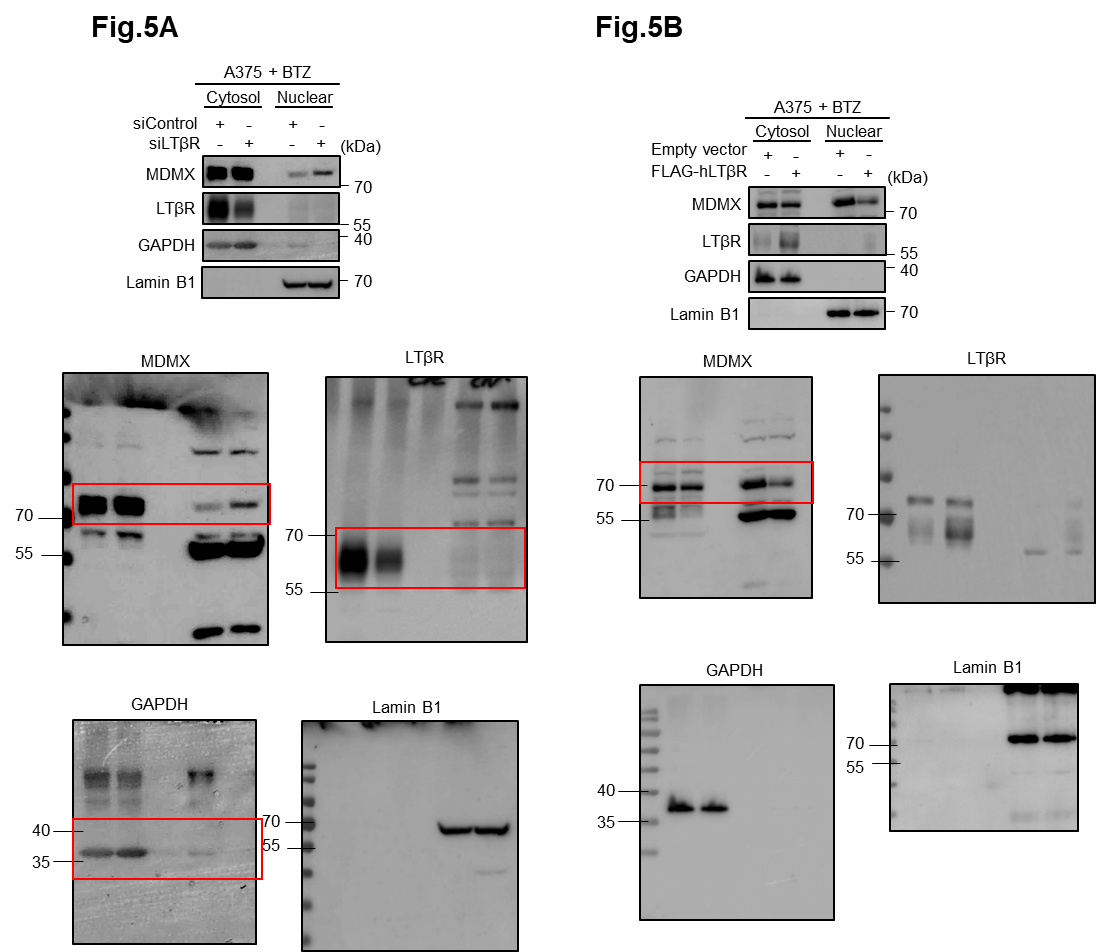


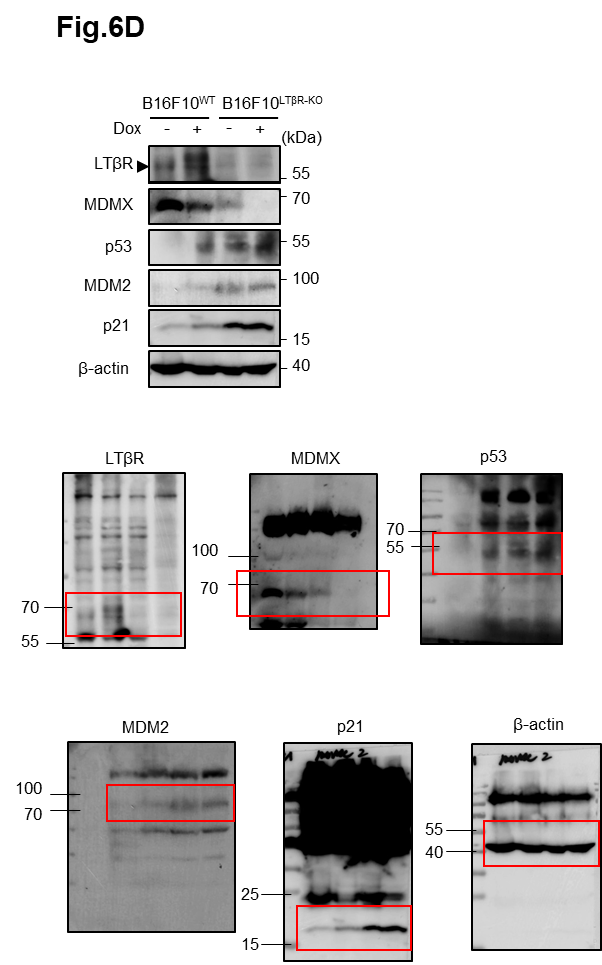


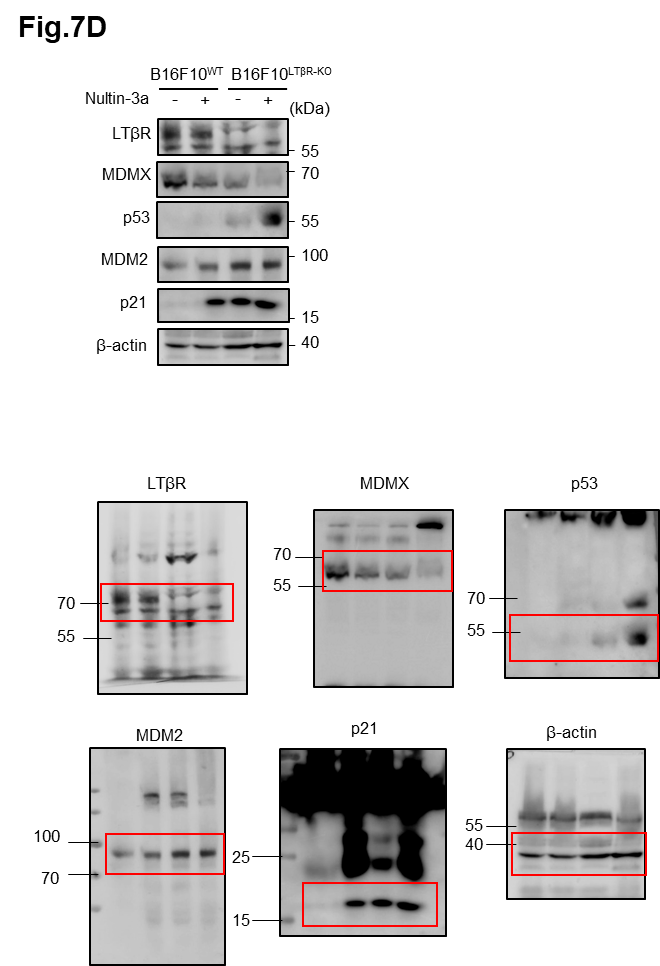


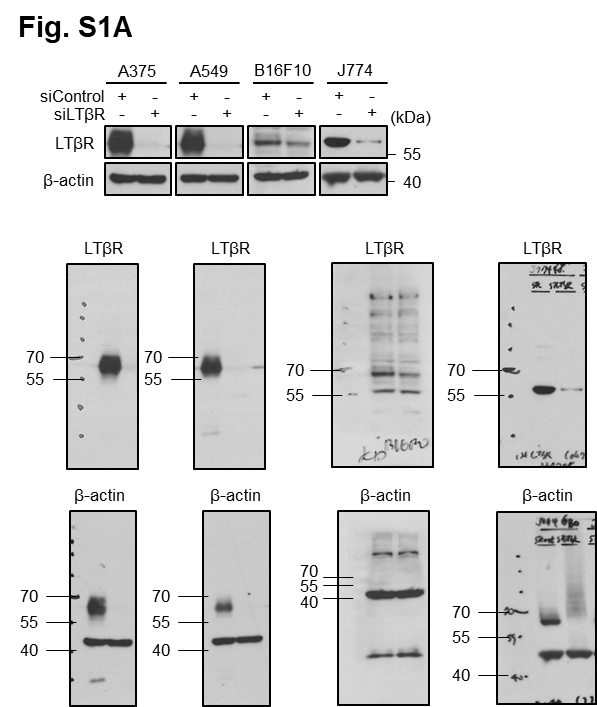


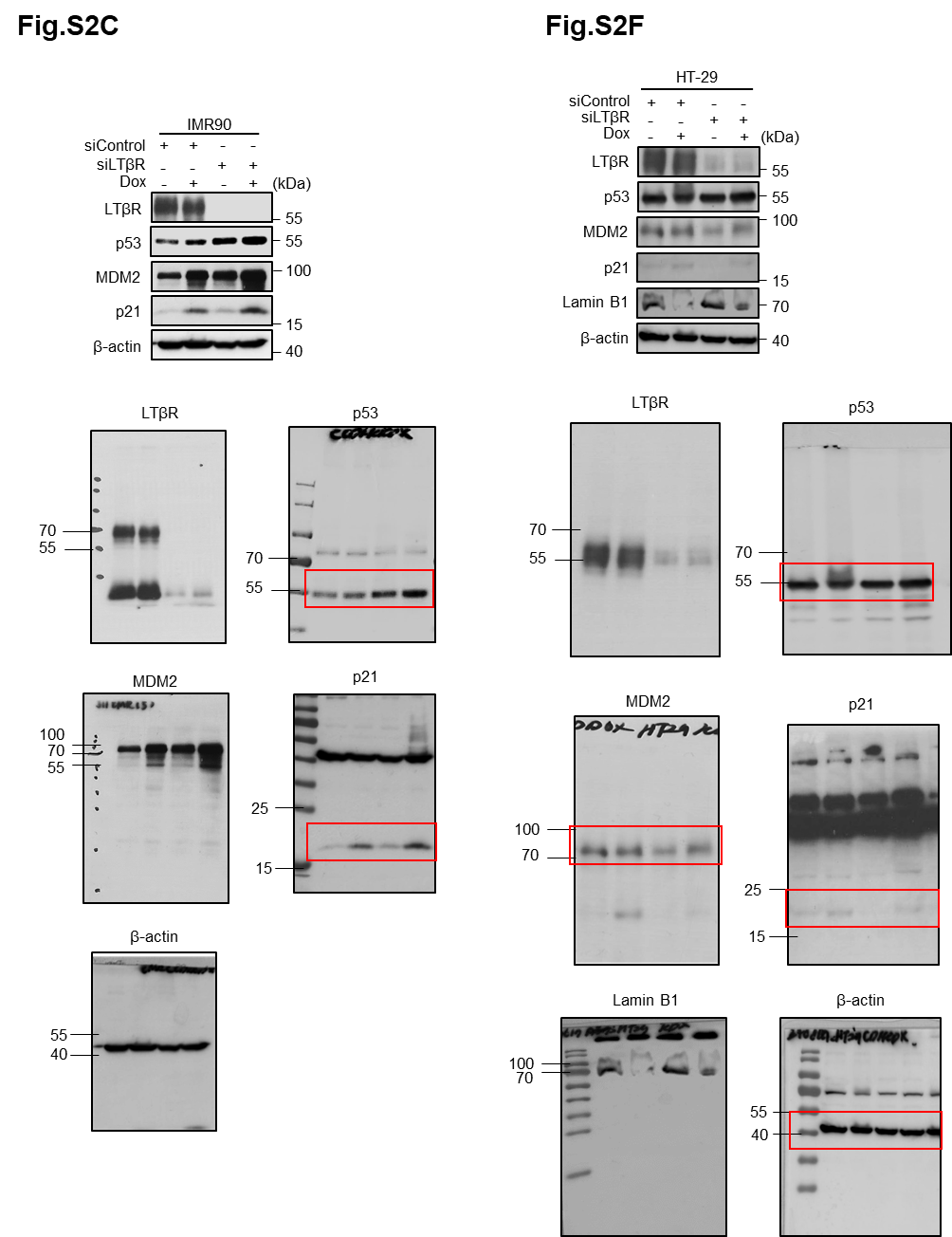


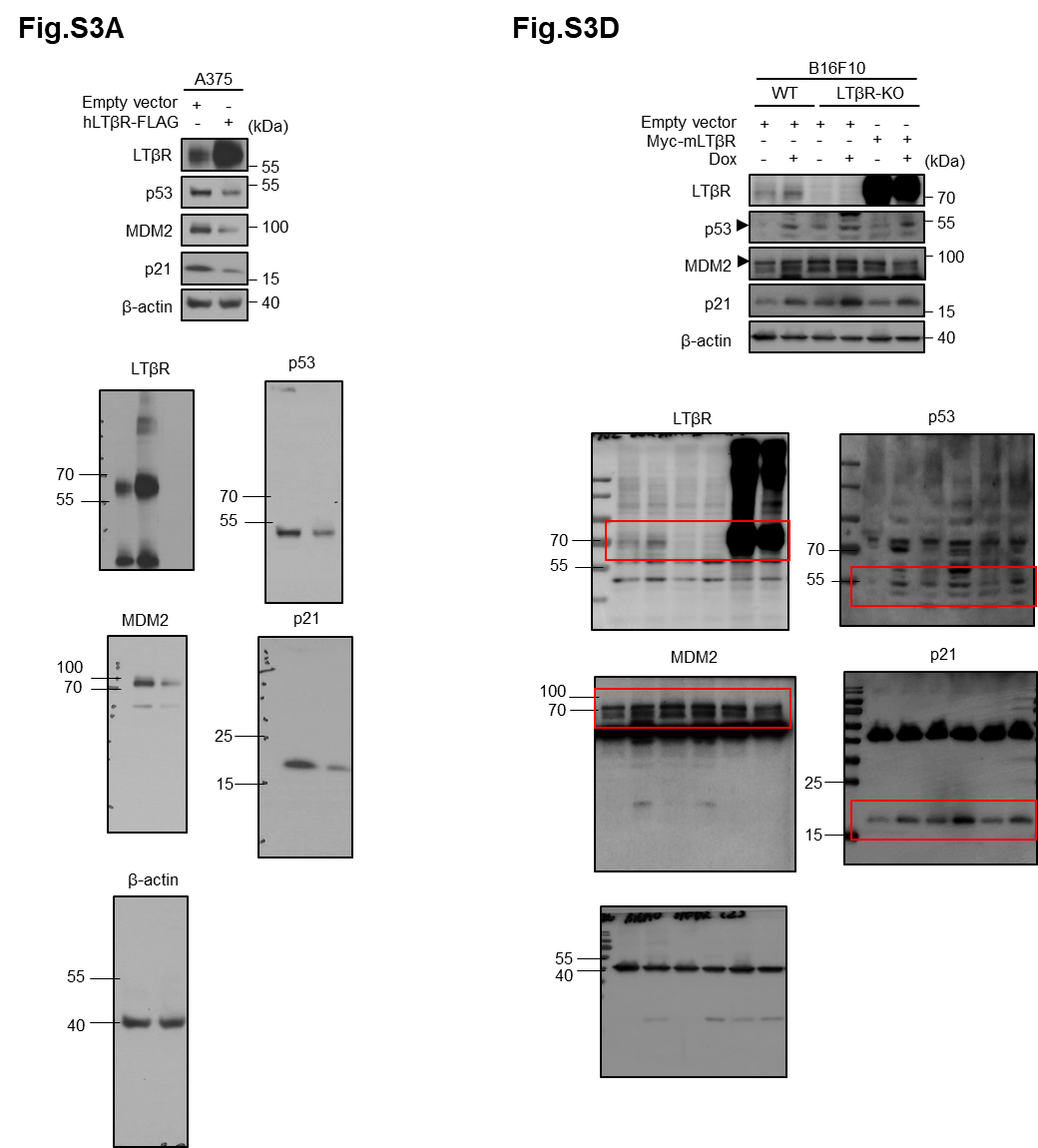


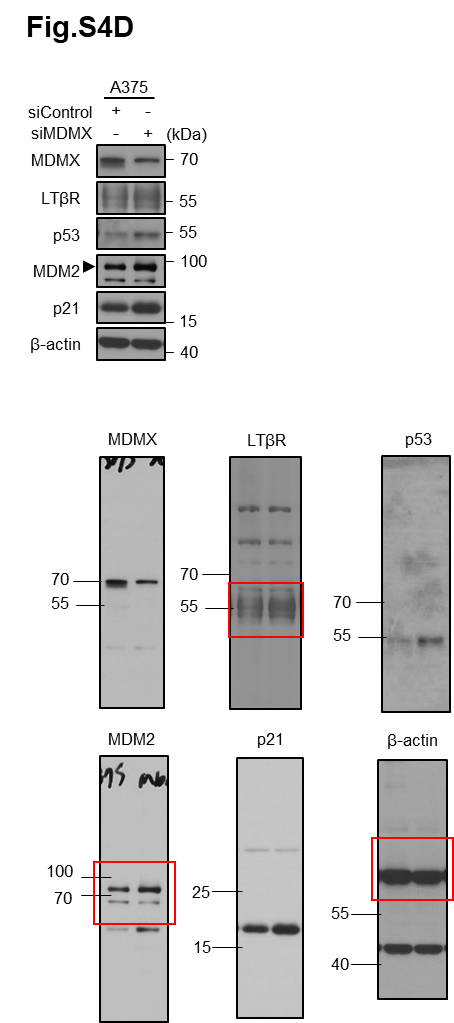


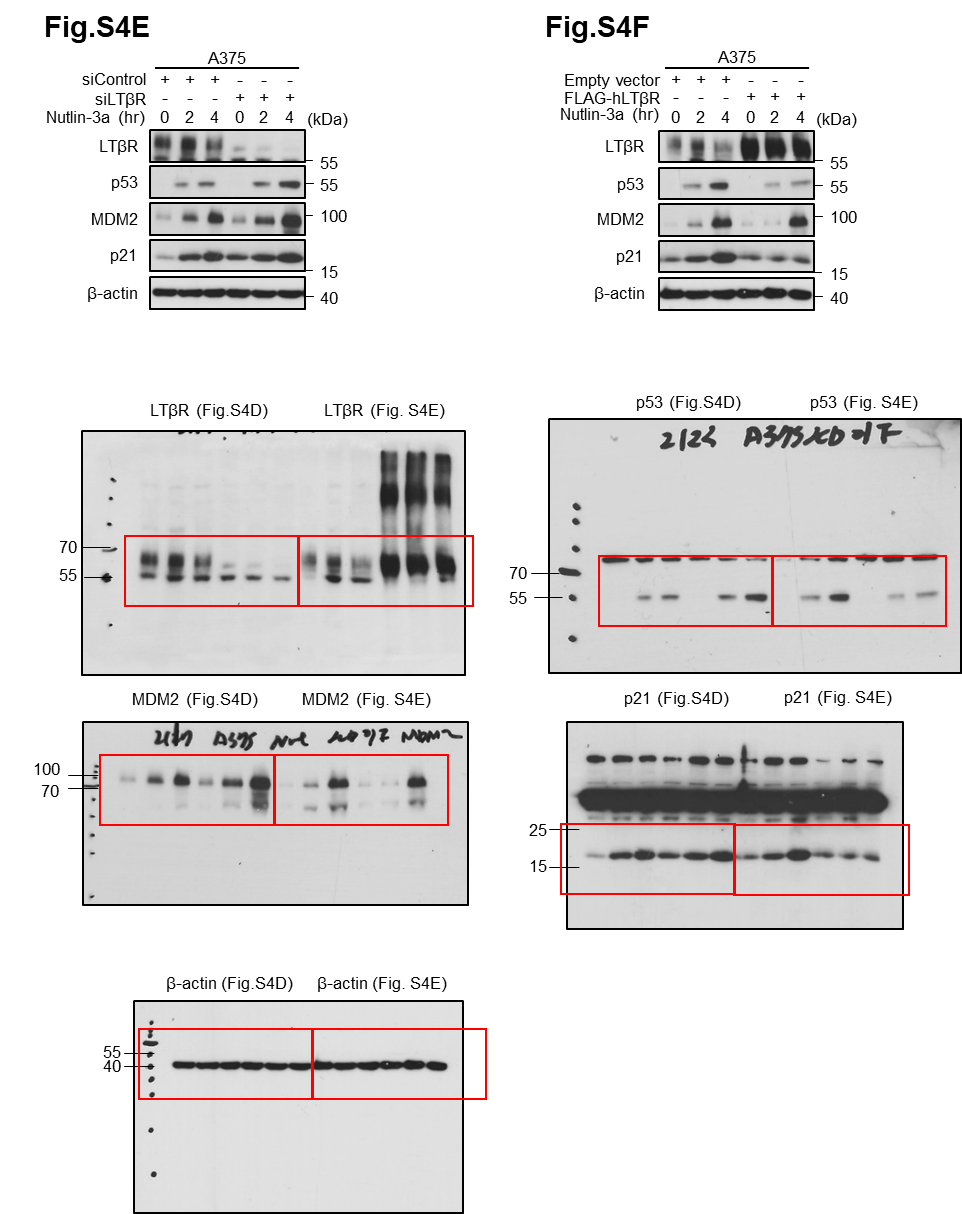


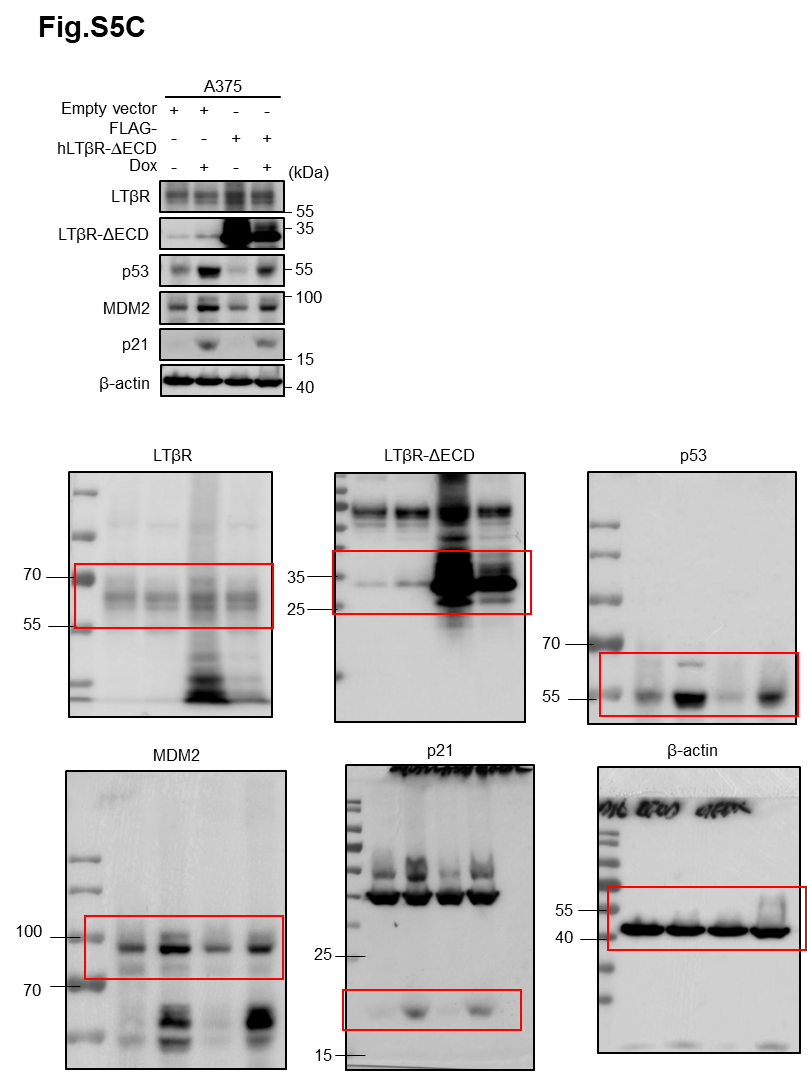


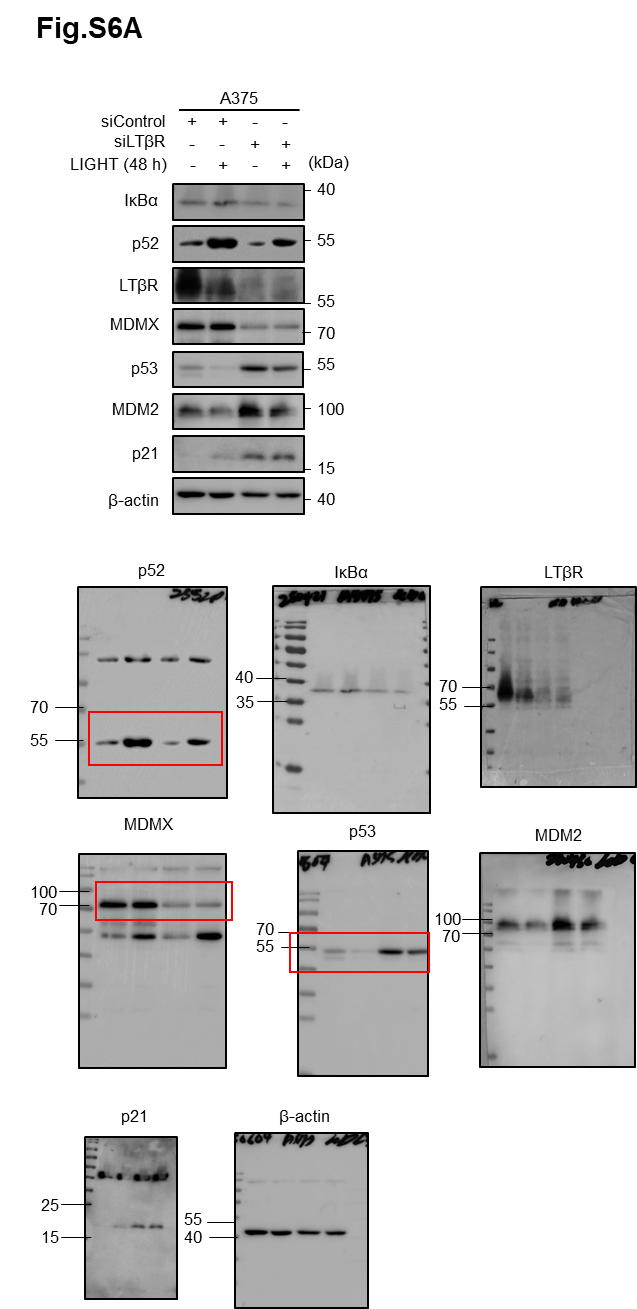


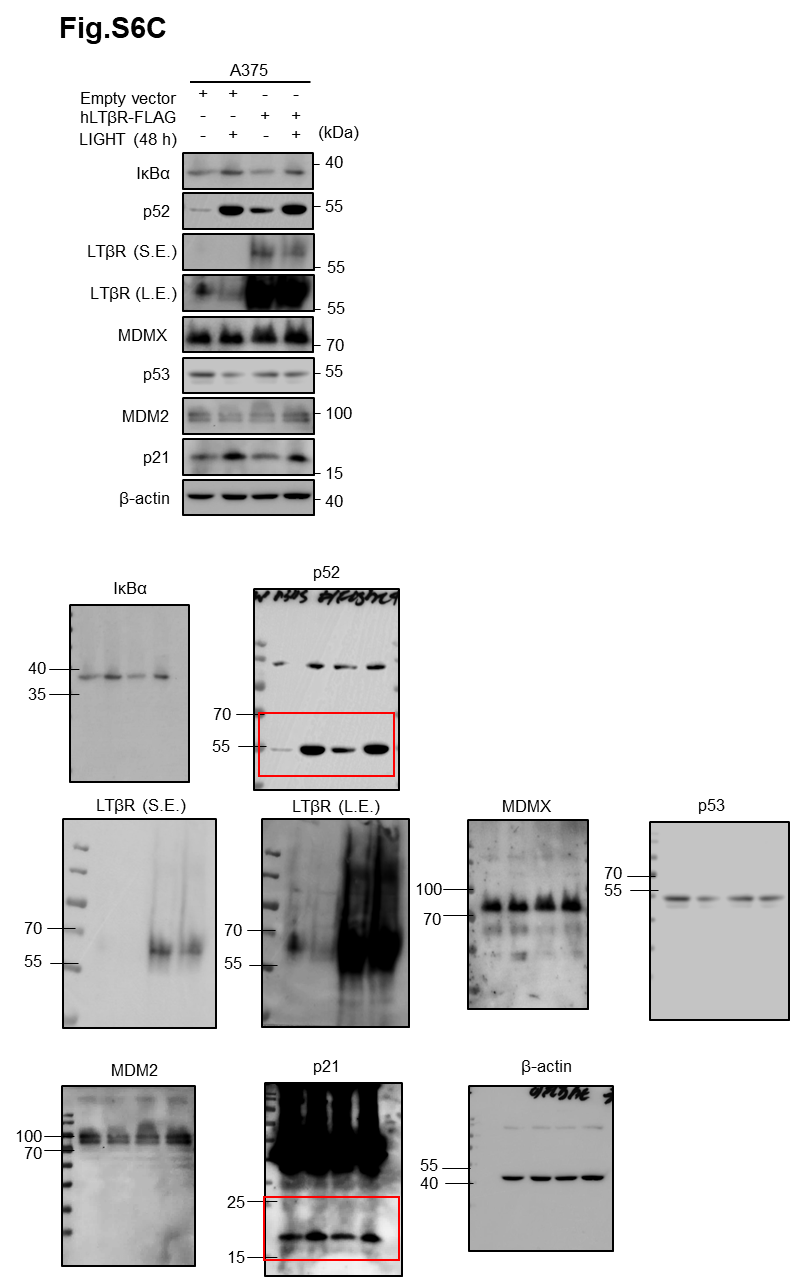


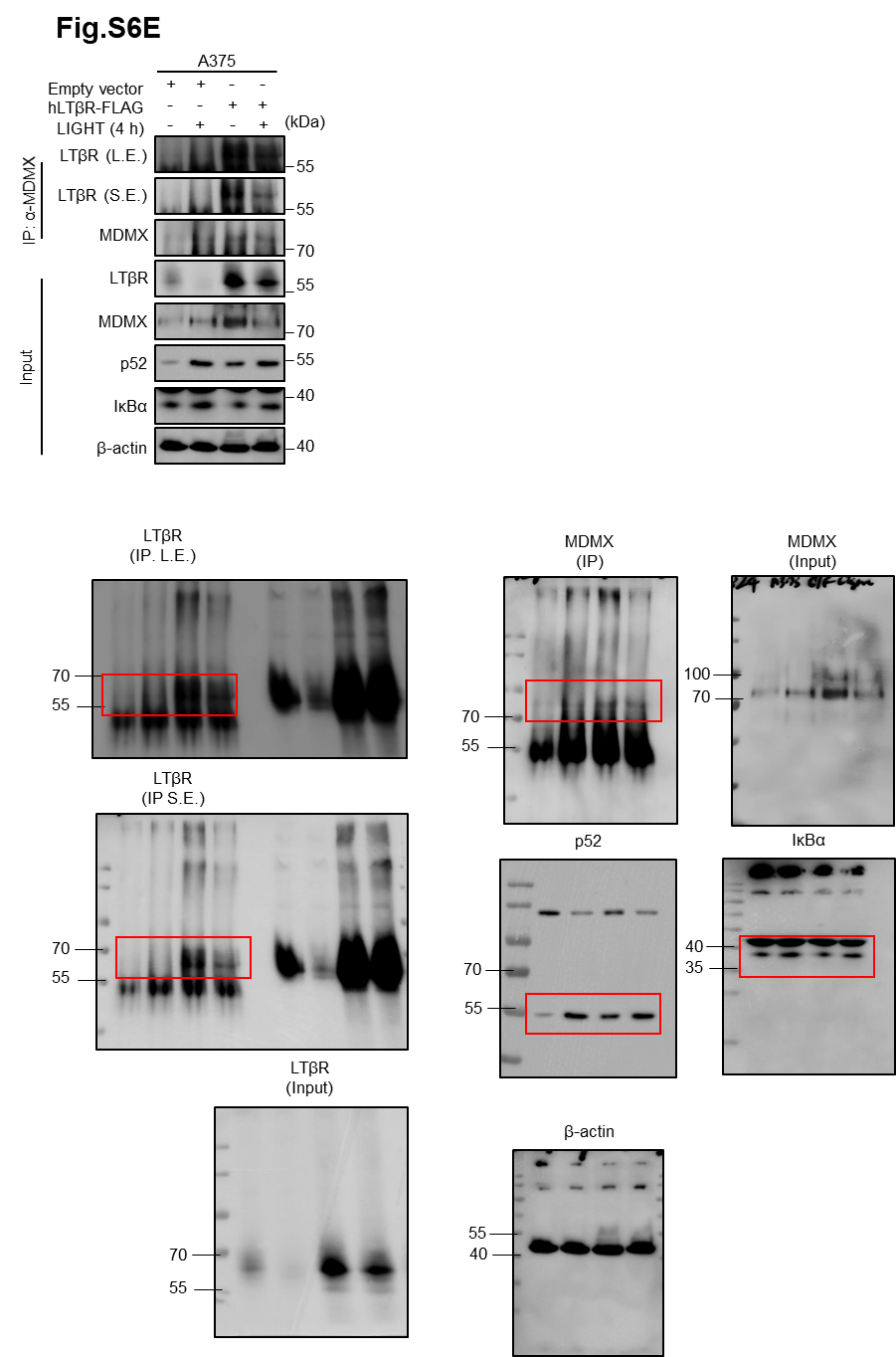


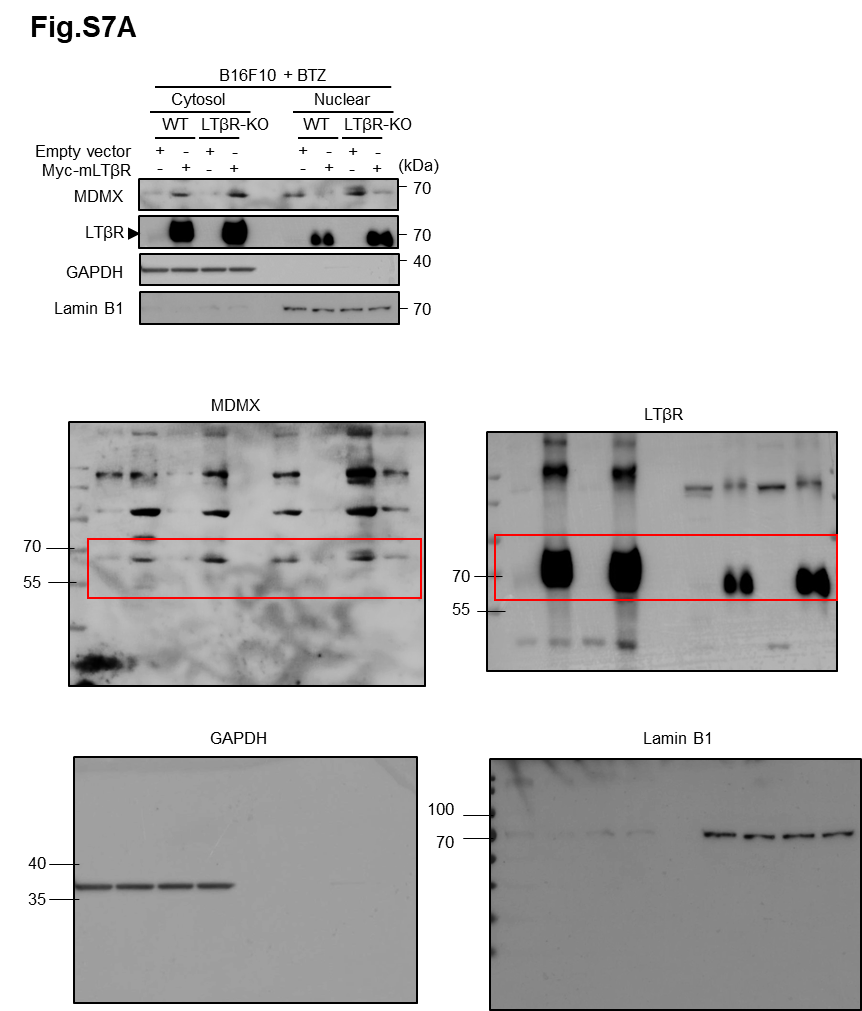


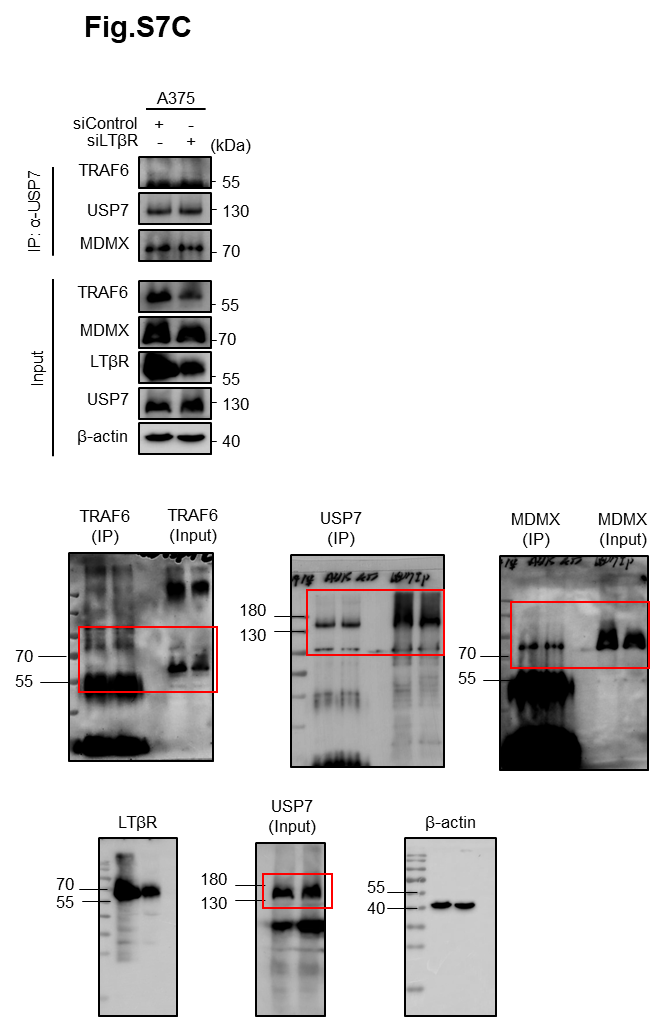


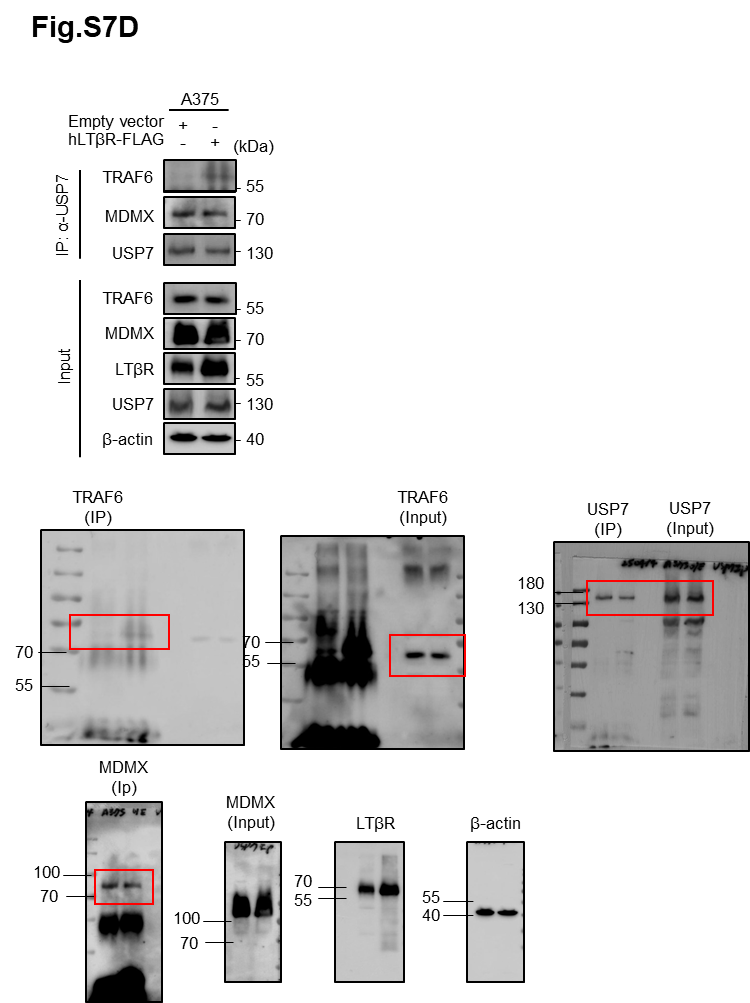

Supplement: Supplementary file 2 — Supplementary Material (Western blot original data) [file 41420_2025_2708_MOESM2_ESM.docx]
